# Top-down modulation of visual processing and knowledge after 250 ms supports object constancy of category decisions

**DOI:** 10.3389/fpsyg.2015.01289

**Published:** 2015-09-16

**Authors:** Haline E. Schendan, Giorgio Ganis

**Affiliations:** ^1^School of Psychology, Cognition Institute, University of PlymouthPlymouth, UK; ^2^Athinoula A. Martinos Center for Biomedical Imaging, Massachusetts General HospitalCharlestown, MA, USA; ^3^Department of Radiology, Harvard Medical SchoolBoston, MA, USA

**Keywords:** category decision, categorization, identification, recognition, object constancy, visual perception, event-related potentials, knowledge memory

## Abstract

People categorize objects more slowly when visual input is highly impoverished instead of optimal. While bottom-up models may explain a decision with optimal input, perceptual hypothesis testing (PHT) theories implicate top-down processes with impoverished input. Brain mechanisms and the time course of PHT are largely unknown. This event-related potential study used a neuroimaging paradigm that implicated prefrontal cortex in top-down modulation of occipitotemporal cortex. Subjects categorized more impoverished and less impoverished real and pseudo objects. PHT theories predict larger impoverishment effects for real than pseudo objects because top-down processes modulate knowledge only for real objects, but different PHT variants predict different timing. Consistent with parietal-prefrontal PHT variants, around 250 ms, the earliest impoverished real object interaction started on an N3 complex, which reflects interactive cortical activity for object cognition. N3 impoverishment effects localized to both prefrontal and occipitotemporal cortex for real objects only. The N3 also showed knowledge effects by 230 ms that localized to occipitotemporal cortex. Later effects reflected (a) word meaning in temporal cortex during the N400, (b) internal evaluation of prior decision and memory processes and secondary higher-order memory involving anterotemporal parts of a default mode network during posterior positivity (P600), and (c) response related activity in posterior cingulate during an anterior slow wave (SW) after 700 ms. Finally, response activity in supplementary motor area during a posterior SW after 900 ms showed impoverishment effects that correlated with RTs. Convergent evidence from studies of vision, memory, and mental imagery which reflects purely top-down inputs, indicates that the N3 reflects the critical top-down processes of PHT. A hybrid multiple-state interactive, PHT and decision theory best explains the visual constancy of object cognition.

## Introduction

People categorize objects accurately (e.g., car, dog, hat) even when visual input is impoverished, for example, due to fog, poor lighting, or unusual viewing angles. They show remarkable visual *constancy* of categorization: People maintain high accuracy despite suboptimal viewing conditions, though performance is slower with impoverished than optimal visual stimuli (Palmer et al., 1981; Tarr et al., 1998). Hierarchical bottom-up processing along the ventral visual stream and frontoparietal decision-making processes have well-established, necessary roles in the visual constancy of category decisions (Tanaka, 2003; Grill-Spector and Malach, 2004; Philiastides and Sajda, 2007). However, recent evidence implicates additional top-down feedback modulations onto posterior information processing areas in order to explain human performance fully, especially under more impoverished conditions (Kosslyn et al., 1994), in which case bottom-up models underperform people (Serre et al., 2007a).

This study aimed to address a critical unanswered issue of when and how bottom-up processes and top-down feedback contribute to visual category decisions. Most prior work focused on functional anatomy using slow hemodynamic measures with a time scale of seconds (Grill-Spector et al., 1999; Lerner et al., 2001), but few used electromagnetic techniques with high time resolution within the range of neural processing (i.e., milliseconds), such as event-related potentials (ERPs), as used here. Also, most studies and theories focus on object cognition under optimal visual input. Consequently, the time when the visual constancy of object cognition is achieved under non-optimal conditions in humans has received relatively little attention.

**Figure 1:**
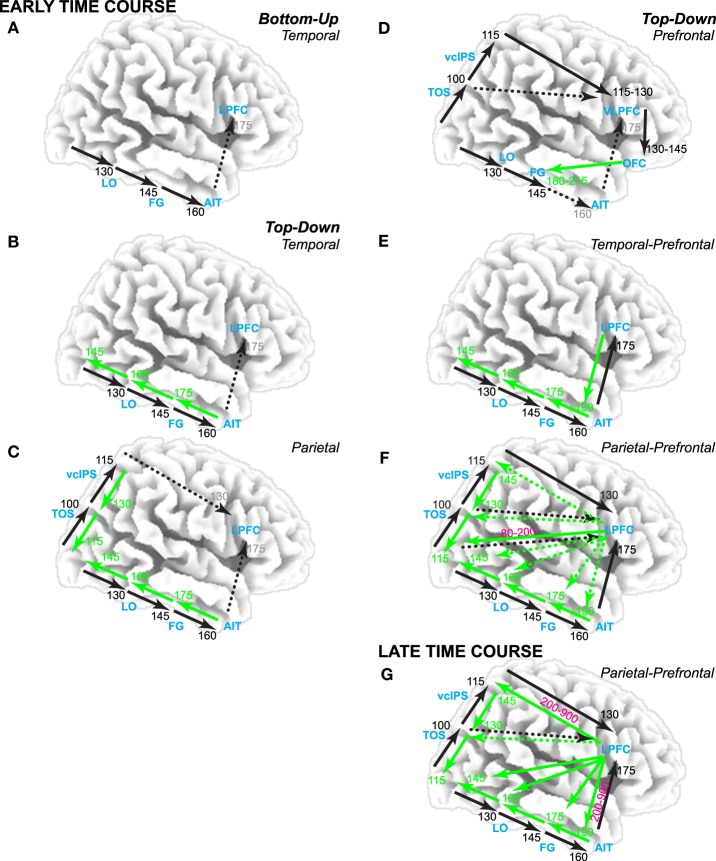
**Theories of visual category decisions**. Timing estimates based on human brain electromagnetic potential data. Black arrows are bottom-up. Green arrows are top-down. Dotted arrows are implied but not specified. Times in black are earliest time of bottom-up input to that region. Times in green are earliest time of feedback input from nearest higher order area to that region. Times in magenta are when prefrontal top-down inputs interact with bottom-up and/or feedback interactions along the visual pathways. Times in gray are associated with implied activity. Theories posit an early time course before 200 ms **(A–F)** or a later time course **(G)**. **(A)** Bottom-up theories posit that the initial feedforward pass through the ventral visual pathway supports object cognition. According to decision theory, this supports a category decision in lateral prefrontal cortex (LPFC). In contrast, *perceptual hypothesis testing* (PHT) theories **(B–G)** emphasize top-down contributions: **(B)** Temporal lobe variants assume bottom-up inputs along the ventral visual hierarchy trigger feedback along the pathway, which consequently modifies bottom-up processing. **(C)** Parietal variants emphasize that the dorsal stream is necessary for complete object constancy. **(D)** One prefrontal variant posits a role for top-down input from ventral LPFC (VLPFC) and orbitofrontal cortex (OFC). **(E)** Temporal-prefrontal variants emphasize bottom-up and feedback processes from visual areas along the ventral pathway through prefrontal cortex. **(F)** Parietal-prefrontal variants emphasize parietal-prefrontal processes of selective attention to locations and features associated with an object category that have been cued by a search template prior to stimulus onset; this modulates visual processing early in time from 80 to 200 ms. **(G)** Late parietal-prefrontal variants emphasize parietal-prefrontal processes of selective attention that contribute model prediction and testing processes when the category is not cued before stimulus onset; note, fMRI tests of parietal-prefrontal PHT variants implicate VLPFC in model prediction and testing (Ganis et al., 2007; Schendan and Stern, 2008).

Timing is important because theories can be grouped into two major classes based on time course, early or late: Early theories propose an early time course within 130–215 ms via bottom-up (Thorpe et al., 1996) and/or top-down processes (Bar, 2003), and late theories propose a later time course and a key role for decision-making (Philiastides and Sajda, 2007) or top-down processes for attention (Stuss et al., 1992; Ganis et al., 2007; Schendan and Lucia, 2010; Clarke et al., 2011). Most vision theories, accounts, or models posit an early time course. Bottom-up models are based on the initial bottom-up pass through the ventral visual hierarchical pathway (Riesenhuber and Poggio, 1999) and posit early time courses (Figure 1A). However, a bottom-up model cannot fully explain the visual constancy of human object cognition (Serre et al., 2007a). For example, on ultra rapid category detection tasks, a name cues the target category before a masked image appears briefly (~20 ms) (Delorme et al., 2000). When masking reduces feedback processing (Di Lollo et al., 2000), the initial fast feedforward sweep along the ventral stream dominates performance, consistent with computational models (Serre et al., 2007a). Critically, however, such bottom-up models cannot match human performance (a) when the mask is removed and so feedback inputs are involved, or (b) when people see the image longer before the mask appears (e.g., 80 vs. 50 ms) because then feedback inputs come into play long enough to boost performance. Bottom-up models also perform poorly when objects are impoverished (as by distance, i.e., farther away). Such limitations led to the suggestion that the bottom-up pathway could provide the initial input and object hypothesis to test using top-down processes (Serre et al., 2007b).

Consequently, other early and late theories posit an important role for feedback inputs. Most of these are *perceptual hypothesis testing* (PHT) theories that propose iterative top-down processes to achieve the visual constancy of object categorization. These top-down processes include *prediction* of a tentative object hypothesis based on prior information (e.g., memory) and *testing* of these predictions using ongoing perceptual input. Top-down processes are important when the stimulus input is ambiguous or impoverished. This is because stimulus ambiguity and impoverishment (e.g., due to rotation, deformation, and illumination changes from one experience to the next) cause the memory and currently perceived object to differ substantially in appearance (Ullman, 1996; Humphreys et al., 1997). This can result in an initial mismatch to stored memory and consequent failure of decision-making processes to categorize the object based on initial bottom-up computations. Temporal lobe, parietal, and prefrontal variants of PHT theories propose different mechanisms.

Temporal lobe variants (Figure 1B) capitalize on reciprocal connections among ventral visual areas in which bottom-up inputs automatically and reflexively trigger feedback from higher-level areas down to lower areas (Bullier, 2001; Ganis and Kosslyn, 2007). In such computational models (Ullman, 1996; Edelman, 1999), higher areas use stored knowledge to reach a fast initial, broad classification that feeds back to lower areas. This first top-down process interacts dynamically with bottom-up perceptual information to refine this classification. A second top-down process uses knowledge about the current context, such as the surrounding scene (e.g., kitchen) or task goal (e.g., find the car), to further select the most appropriate object model to feedback to lower level areas (Ullman, 1996). In addition, reverse hierarchy theory (Hochstein and Ahissar, 2002) proposes further that, once the initial bottom-up pass reaches advanced ventral visual areas, top-down processes for selective attention bind sensory features, and conscious visual perception begins (Treisman, 2006). Consequently, perceptual hypotheses are generated that project back along the visual hierarchy in reverse order to lower-level areas, which provide the detailed information needed to test the hypotheses. Interactive activation and competition theory (Humphreys et al., 1995, 1999) proposes further that these processes are task-dependent (e.g., most important for object naming) and involve multiple knowledge stores, which are themselves connected recurrently within and between each other (Price et al., 1996): A structural description system in left posterior inferotemporal cortex stores knowledge about shape and interacts with a semantic memory system, which, in turn, interacts with knowledge systems that store the names and semantic classes (e.g., animal, vehicle, tool).

Parietal and parietal-prefrontal variants propose that the ventral stream can support decisions about an object from known views, but, when viewing an object from an angle that impoverishes the image, additional spatial transformations must be computed, such as those implicated in mental rotation (Tarr and Pinker, 1989; Turnbull et al., 1997; Gauthier et al., 2002). These transforms align the percept and stored object knowledge spatially (Bülthoff et al., 1995) and may be implemented in occipitoparietal areas along the dorsal visual stream. A parietal variant predicts dorsal transforms are rapid, happening within 200 ms (Figure 1C), because the dorsal stream processes visual information faster than the ventral stream (Bullier, 2001). A parietal-prefrontal variant involves mental imagery processes implicated in mental rotation, which are slow because they involve top-down processes from prefrontal cortex after 200 or 500 ms that are implicated in selective attention and model verification (see parietal-prefrontal theories below, Figure 1G) (Schendan and Kutas, 2003; Schendan and Lucia, 2009).

While temporal and parietal variants imply a role for prefrontal cortex, prefrontal variants specify such a role. One prefrontal variant (Figure 1D) assumes that people routinely accomplish object cognition within about 200 ms using low spatial frequency information from V2/V4 to compute a coarse scene representation along the dorsal pathway (Bar, 2003). This representation is sent forward rapidly into Brodmann's area (BA) 45 of ventral lateral prefrontal cortex (VLPFC) and then orbitofrontal cortex, which uses this information to predict possible categories within 130 ms after visual stimulation and feeds these back to fusiform cortex in the ventral stream within 180–215 ms (Bar et al., 2006). Other prefrontal PHT variants can be summarized within a free-energy type framework (Friston, 2010). Of these, temporal-prefrontal variants focus on ventral stream and prefrontal interactions (Figure 1E). For example, in hierarchical Bayesian models (Lee and Mumford, 2003), bottom-up processes (e.g., ventral stream) can yield a perceptual hypothesis that serves as a predictive code to test using information coming in from the stimulus (e.g., to prefrontal cortex). In contrast, parietal-prefrontal variants implicate top-down selective attention processes, which involve interactions between parietal and prefrontal cortex (Spreng et al., 2013). For example, in one such variant (Figure 1F), dorsolateral prefrontal area 46 feeds back a signal to visual areas that competitively biases processing of features at the attended location that match the search template for the object (Deco and Rolls, 2004). Spatial biases feedback via the dorsal pathway, and object biases feedback via the ventral pathway. This model aims to explain cognition when the location or object is cued before the stimulus and so attention can modulate early visual processing within 200 ms (Di Russo et al., 2003). In contrast, other models explain category decisions without cueing and implicate processes primarily after the initial bottom-up activation of the ventral stream, that is, after 200 ms (Figure 1G). For example, model verification theory (Lowe, 2000) proposes that, for a slightly impoverished image, the bottom-up pass can suffice to match the percept to the correct model, whereas for a more impoverished image (e.g., degraded picture), the bottom-up pass may only find a weak match to knowledge (or initial classification Ullman, 1996) that is insufficient for an accurate decision. Consequently, top-down processes implicated in selective attention perform model verification to determine the knowledge in posterior cortex that best explains the percept. A prediction process selects the locations of salient features, evaluates their match to knowledge, and generates a prediction about a candidate object model (e.g., a category). A testing process, which may involve parietal spatial transformation and mental rotation processes (e.g., as in some parietal vision theories), evaluates the predicted model for its fit with the percept. An adaptive resonance variant provides important computational solutions for how such processes may operate (Fazl et al., 2009), such as a mismatch reset signal from prefrontal cortex that controls prediction and testing cycles until enough evidence accumulates for a decision.

While vision and decision theories have evolved separately, both explain category decisions under uncertainty due to impoverished sensory input, and decision theories specify roles for prefrontal and parietal cortex. Evidence accumulation is a core process in decision-making theories (Ratcliff, 1978), which offer mathematical solutions for how frontoparietal areas accumulate and evaluate evidence for a decision (Gold and Shadlen, 2007). As perceptual impoverishment increases, decision certainty decreases, and decision processes are recruited more. Decision theories explain decision processes based on information from perception (Gold and Shadlen, 2007), category knowledge (Philiastides and Sajda, 2007), and recognition memory (Ratcliff, 1978). Decision accounts propose that prefrontal and parietal cortices accumulate evidence from ventral areas via bottom-up inputs (Philiastides and Sajda, 2007), making them bottom-up theories (like Figure 1A or the bottom-up pathways in Figure 1F). Critically, the brain regions and event-related potentials (ERPs) associated with category decisions and impoverishment effects on visual cognition are similar (e.g., Schendan and Kutas, 2003; Ganis et al., 2007; Jiang et al., 2007; Schendan and Stern, 2008; Wheeler et al., 2008). Findings from the present study favor a hybrid decision and parietal-prefrontal PHT theory in which both bottom-up and top-down interactions occur between prefrontal decision and posterior evidence components of the brain's decision network (Figure 1G).

In summary (Figure 1), vision and decision theories differ in involvement of parietal and prefrontal cortex and various top-down processes, which predicts different time courses. All propose object constancy of category decisions within 200 ms, except for parietal-prefrontal PHT theories that propose that, when the category is unknown before stimulus onset, interactive bottom-up and feedback processes from the visual pathways into lateral prefrontal cortex between 200 and 900 ms support object constancy.

The present study aimed to define the time course of category decisions under uncertainty due to impoverished visual input. To do so, ERPs were recorded using the paradigm from an fMRI study (Ganis et al., 2007) that uniquely manipulated both visual impoverishment and knowledge and found evidence favoring parietal-prefrontal PHT and decision theories (Philiastides and Sajda, 2007). Subjects decided whether they could categorize more (MI) and less (LI) impoverished drawings of real objects and pseudo versions of them, which differ in knowledge activation. FMRI activation is greater for MI than LI images, and more so for real than pseudo objects in the VLPFC (BA 45 and 47/12), occipitoparietal, and occipitotemporal object processing areas implicated in selective attention, spatial transformation, and category decisions. Critically, this *impoverished-real-object effect* implicates not only perceptual processing but also the knowledge activation needed for PHT and a category decision. After all, by design, real objects activate knowledge, whereas the novel shapes of pseudo objects do so minimally if at all (Kroll and Potter, 1984). Thus, impoverishment effects for both object types reveal perceptual processing, whereas those for real more than pseudo objects reflect knowledge processing, thereby distinguishing between the contributions of sensory-perceptual vs. knowledge (i.e., memory) evidence used for PHT and a category decision. Critically, the fMRI pattern for impoverished real objects refutes a purely bottom-up account of object constancy, which predicts the opposite impoverishment effect (i.e., greater activation for LI images, regardless of object type, because LI images have more perceptual features). Moreover, when top-down processes for visuospatial working memory cannot be engaged fully in a category decision, performance is impaired with MI (but not LI) objects (Ganis et al., 2007). Thus, altogether, convergent evidence indicates that impoverished-real-object effects reflect top-down contributions, not only bottom-up input, to PHT and category decisions.

This design improves upon electromagnetic brain potential studies on object constancy, decisions, and category knowledge in four ways as follows. (1) It manipulates both impoverishment and object type (i.e., knowledge). Previously, either impoverishment of real objects in fragmented drawings (Viggiano and Kutas, 2000; Schendan and Kutas, 2002, 2007a; Schendan and Maher, 2009) and rotated views varied (Schendan and Kutas, 2003) or categorization success (knowledge) varied between stimuli (Holcomb and McPherson, 1994; Schendan et al., 1998; McPherson and Holcomb, 1999; Gruber and Müller, 2005, 2006; Gruber et al., 2006; Sehatpour et al., 2006, 2008; Schendan and Maher, 2009; Voss et al., 2010). (2) Pseudo objects here had been constructed from the real objects to equate them on low-level features, perceptual properties, and coherent object structure, and, in work with these intact versions, ERPs differ only after 175 ms when initial bottom-up processing is largely complete, confirming matched low-level sensory attributes between types (Schendan et al., 1998). Other studies compared real objects relative to either pseudo objects chosen from a different set of real objects that were unknown to subjects (Holcomb and McPherson, 1994; McPherson and Holcomb, 1999) or distorted or scrambled versions that are unknown (Gruber and Müller, 2005, 2006; Busch et al., 2006; Gruber et al., 2006; Sehatpour et al., 2006, 2008), or compared objects with less than more novel or meaningful visual structures (Daffner et al., 2000a; Folstein and van Petten, 2008; Voss et al., 2010). Notably, despite these visual differences, all these studies confirm ERP effects only after 175 or 215 ms, suggesting that knowledge is the primary factor distinguishing real and pseudo objects. (3) This experiment assessed many categories, whereas ERP work on category decisions focused on face-selective activity with cars as the comparison category (Philiastides et al., 2006; Philiastides and Sajda, 2006, 2007). (4) There is no repetition confound. Here, subjects categorize each object once, instead of repeatedly at multiple levels of impoverishment (Stuss et al., 1986; Doniger et al., 2000; Viggiano and Kutas, 2000; Schendan and Kutas, 2002; Philiastides and Sajda, 2006; Ratcliff et al., 2009). This is important because repetition affects behavior (i.e., priming) and ERPs, making them more positive after 200 ms (Schendan and Kutas, 2003, 2007a; Henson et al., 2004; Schendan and Maher, 2009), and these effects are larger for meaningful than meaningless objects (e.g., real vs. pseudo) (Snodgrass and Feenan, 1990; Schendan and Kutas, 2002; Schendan and Maher, 2009; Voss et al., 2010). Further, repetition effects differ between impoverishment levels, being largest at moderate levels (Snodgrass and Feenan, 1990) and when objects repeat from LI to MI than MI to LI (Schendan and Kutas, 2003).

The time when ERPs show the impoverished-real-object effect defines when PHT and decision processes contribute to the visual constancy of category decisions based on knowledge, not just sensory evidence. To infer the timing of cortical sources, ERP results were integrated with fMRI location information by both estimating the ERP sources and relating similar functional patterns between methods (Luck, 1999). To use vision and decision theories to predict the ERP effects, this report capitalizes on the multiple-state interactive (MUSI) account of the brain basis of visual object cognition to define the times and scalp sites to analyze (Schendan and Kutas, 2003, 2007a; Schendan and Maher, 2009; Schendan and Ganis, 2012). This framework proposes that posterior object processing areas activate at multiple times in brain “states” serving distinct functions. This account extends the principle that different brain areas can perform different functions for cognition at different points in time because bottom-up, feedback, and recurrent activity alters neuronal computations, as demonstrated, for example, in visual area V1 (Lamme and Roelfsema, 2000). Likewise, object-sensitive areas perform different functions in perception and cognition due to different neural computations associated with bottom-up, feedback, and recurrent activity (Schendan and Lucia, 2010).

**Table:** 

State 1:	Initial activity in object processing areas feeds forward from occipital to temporal cortex between ~120 and ~200 ms when a visual object is broadly perceptually categorized (e.g., as a face instead of nonface object) (Schendan et al., 1998; Schendan and Ganis, 2013), as described for ventral visual hierarchy processing (Figures 1A,B, 11). This state is indexed by early ERPs reflecting activity in object-sensitive areas related to categorical perception: the vertex positive potential and its occipitotemporal N170 counterpart (VPP/N170) (Schendan and Lucia, 2010). When input is optimal, this predominantly bottom-up activation of knowledge should be sufficient (Serre et al., 2007a) to enable object cognition (i.e., entry level categorization) and phenomenological awareness of this knowledge in State 2 with little or no need for additional top-down processing from prefrontal cortex (Schendan and Kutas, 2007a).
State 2:	Object processing areas activate again interactively due primarily to top-down processing among these areas and VLPFC as well as other areas such as parietal cortex (Schendan and Lucia, 2009). This is indexed by mid-latency negative ERPs between 200 and 500 ms: an N3 complex (including components known as template matching N2[00], N300, N350, frontal N400). The N3 is the first ERP in response to pictures that modulates according to cognitive factors affecting posterior object processing cortex and VLPFC similarly (Barrett and Rugg, 1990; Zhang et al., 1995; McPherson and Holcomb, 1999; Doniger et al., 2000, 2001; Curran et al., 2002; Schendan and Kutas, 2002, 2003, 2007a; Folstein and van Petten, 2004, 2008; Philiastides and Sajda, 2006, 2007; Philiastides et al., 2006; Sehatpour et al., 2006; Gratton et al., 2009; Schendan and Lucia, 2009, 2010) and that localizes to these brain areas (David et al., 2005, 2006; Sehatpour et al., 2008; Schendan and Maher, 2009; Schendan and Lucia, 2010; Clarke et al., 2011; Bastin et al., 2013). States 1 and 2 are thus described in the time course for late parietal-prefrontal PHT theories (Figure 1G) and are consistent with these ideas for the first 500 ms of visual processing.
State 3:	Top-down interactive processes, including conscious, effortful, cognitive control functions, perform internal evaluation, and verification after about 400 to 500 ms. For example, (a) a parietal P600 (or P3[00]) component reflects later strategic evaluation or verification of earlier category decision processes, being more positive for correct decisions, and strategic, effortful mental rotation of objects, being larger when more mental rotation is needed, and (b) a parietal late positive complex (LPC) complex is associated with higher-order semantic analysis, being larger when semantic integration is more challenging (i.e., contextually incongruous) (Schendan and Lucia, 2009; Schendan and Maher, 2009; Sitnikova et al., 2010).

For each theory, Table 1 summarizes the predictions for the pattern of ERP effects, and the MUSI framework specifies the ERPs, effects, and their direction. Posterior cortex theories (Figures 1A–C) predict only early effects. See Table 1 (VPP/N170 predictions i): All vision theories in Figure 1 predict the same impoverishment and type effects between 130 and 215 ms. This is explained by the bottom-up processes in these theories. Bottom-up processing (e.g., Figure 1A) predicts overall less neural activity for MI than LI objects and for pseudo than real objects (i.e., independent impoverishment and type effects) during the initial bottom-up pass through the ventral stream in state 1. The impoverishment effect happens because MI objects show fewer visual features and so they activate fewer neurons and/or activate each neuron less, relative to LI objects. The type effect happens because the initial pass categorizes by activating knowledge, which is less successful for pseudo than real objects, by design. Altogether, this predicts that the VPP/N170 will be larger for LI than MI and for real than pseudo objects (see Table 1 Bottom-up).

**Table 1:** **Predicted pattern of impoverishment (I) and type (T) effects according to vision and decision theories and summary of ERP results**.

**Predictions**				**Theory**					**Results**
	**ERP**	**Effect**	**Direction**	**Bottom–up**	**Top–down perceptual hypothesis testing/decision**				
				**Early**	**Early**		**Late**		
				**Temporal**	**Temporal and Parietal**	**Prefrontal**	**Parietal–prefrontal**	**MUSI and Decision**	
i	VPP/N170	I	LI Larger	X	X	X	X	–	–
i	145–160 ms	T	Real larger	X	X	X	X	–	X?
ii		I × T	I Larger for real	–	X	X	–	–	–
iii	N3	I	MI larger	–	–	?	X	X	X
iii	200–500 ms	T	Real larger	–	–	?	X	X	X
iv		I × T	I larger for real	–	–	–	X	?	X
iii	P600	I	LI larger	–	–	?	X	X	X
iii	500–900 ms	T	Real larger	–	–	?	X	X	X
iv		I × T	I Larger for real	–	–	–	X	?	X
	SW	I	LI larger						X
	700–900 ms	T	Real larger						X
		I × T	–						X
				Figure 1A	Figures 1B,C	Figures 1D–F	Figure 1G	Figures 1G, 11	Figure 11

*LI, less impoverished; MI, More impoverished; MUSI, Multiple State Interactive account of visual object cognition; X, predicted effect; ?, consistent but not specifically predicted; X?, Spurious effect due to low level sensory differences. N400 (300–500 ms) predictions and results are the same as for the N3. LPC (late positive complex) predictions and results are the same as for the P600. Early prefrontal theories include early prefrontal, temporal-prefrontal, and parietal-prefrontal as in Figures 1D–F*.

See Table 1 (predictions ii): Temporal, parietal, and prefrontal variants of top-down PHT theories (Figures 1B–D, respectively) predict, in addition, early impoverished-real-object effects (see Table 1 Temporal and Parietal, and Prefrontal) due to feedback at this time; note, for one prefrontal variant (Bar, 2003), this interaction effect will be found as long as MI stimuli contain sufficient low spatial frequency information to compute a coarse object representation along the dorsal stream.

See Table 1 (Prefrontal; predictions iii): Prefrontal PHT variants can accommodate (Figures 1D–F) or predict (Figure 1G) later type and impoverishment effects. For example, one early prefrontal PHT variant can accommodate additional late type and impoverishment effects (see bottom-up inputs to AIT and VLPFC in Figure 1D). Type effects occur at later times when meaning is activated after categorization (Bar et al., 2006). Also later during post-categorization times, high spatial frequencies have a role (Bar, 2003), predicting impoverishment effects at later times due to less power at high spatial frequencies in MI than LI pictures. Early temporal-prefrontal and parietal-prefrontal PHT variants (Figures 1E,F) can likewise accommodate late type and impoverishment effects based on post-categorization processes. However, as categorization is already done, none of these predict late impoverished-real-object effects. Only late parietal-prefrontal PHT theories predict late type and impoverishment effects, as these propose that knowledge activation for the category decision with MI objects continues to be attempted after the initial bottom-up pass, that is, after 200 ms. The MUSI framework (Table 1) predicts the direction of these late ERP effects. Late ERPs will be more negative for MI than LI stimuli (impoverishment effect) and for real than pseudo objects (type effect); in other words, the N3 will be larger for MI stimuli and pseudo objects, whereas the P600/LPC will be larger for LI stimuli and real objects. This is due to stronger activation of memory for real than pseudo objects and LI than MI stimuli. This direction of impoverishment effects on the P600/LPC is also predicted by the slow mental rotation process in some parietal-prefrontal PHT variants (Figure 1G) because negativity is greater for more than less rotated objects (i.e., impoverished regarding match to memory) during mental rotation (Schendan and Lucia, 2009).

See Table 1 (predictions iv): Late parietal-prefrontal PHT variants (Figure 1G) assume that bottom-up processing before 200 ms (as in Figure 1A) provides the front-end to later top-down processes, which predict later impoverished-real-object effects after 200 ms. The interaction effect would happen when prefrontal cortex biases attention (Deco and Rolls, 2004) or uses attention processes to control prediction and testing cycles (Lowe, 2000; Fazl et al., 2009). A later time course is consistent with ERP evidence for feature search along the ventral stream between 150–200 and 300–450 ms (Luck, 2006). By some accounts, the interaction happens when late mental rotation processes in frontoparietal cortex are recruited (Tarr and Pinker, 1989; Schendan and Stern, 2008). This predicts the interaction after 200 ms in state 2 during the N3 when parietal feedback interactions compute spatial relations among object parts and, especially after ~500 ms in state 3 during the P600/LPC when spatial transformations implicated in mental imagery of object rotation happen (Schendan and Lucia, 2009). Note, some temporal-prefrontal PHT variants (Figure 1E, Humphreys et al., 1997; Hochstein and Ahissar, 2002) and decision theories can suggest an add-on of later selective attention processes that would essentially be the same mechanism described in parietal-prefrontal PHT theories (Figure 1G) and so could accommodate late type and impoverishment effects and their interaction. In addition, because these theories use a bottom-up model as the front end to hypothesis testing (e.g., model verification) or decision processes, they predict the same pattern of early effects as bottom-up models: Early impoverishment and type effects. They also predict no early interaction effects because frontoparietal contributions happen later.

The MUSI framework and decision theories predict type and impoverishment effects only during later ERPs. MUSI predicts this because category decision processes happen after the initial bottom-up pass after 200 ms (Schendan and Maher, 2009). Decision theories predict this due to bottom-up accumulation of evidence in frontoparietal areas implicated in decision-making and task difficulty between 200 and 450 ms during the D220 and late component (Philiastides and Sajda, 2007), which correspond to components of the N3 complex. MUSI and decision theories do not predict but can accommodate late impoverished-real-object effects, as both posit late prefrontal activity, and MUSI posits further that prefrontal top-down processes are critical for category decisions. Finally, note, most vision theories, other than parietal and parietal-prefrontal PHT theories, were created to explain cognition with optimal input so are problematic for predicting effects with MI stimuli and pseudo objects, but it is important to attempt to make explicit predictions in order to test the strengths and limitations of these theories.

For completeness, we assessed two other late ERPs that modulate during category decisions. Later in state 2, the centroparietal N400 between 300 and 500 ms reflects interactive activation of semantic memory, especially meaningful knowledge associated with linguistic stimuli (e.g., a name), in anterior temporal cortex and VLPFC (Marinkovic et al., 2003; Lau et al., 2008; Kutas and Federmeier, 2011). Only parietal-prefrontal PHT and decision theories posit a role for word meaning, which is knowledge that can contribute to category decisions and prediction. Hence, the N400 will be more negative for MI than LI and for real than pseudo objects and show impoverished-real-object effects, like the N3 and P600/LPC (Table 1). Also, a broad slow wave (SW) starting around 700 ms has been associated with response planning for category decisions, including naming, being more positive for named than unnamed objects (Schendan and Kutas, 2002, 2003; Folstein et al., 2008; Schendan and Lucia, 2009; Schendan and Maher, 2009; Sitnikova et al., 2010). This predicts greater SW positivity for LI than MI and for real than pseudo objects, but no interaction, as the SW reflects processes after the category decision.

## Materials and methods

Methods were the same as for the event-related fMRI version (Ganis et al., 2007) except for modifications needed for ERPs.

### Materials

Fragmented drawings from the Snodgrass and Vanderwart (1980) set depicted 128 real objects and 64 pseudo versions of them. For a prior ERP study (Schendan et al., 1998), we created pseudo objects by rearranging parts of the real objects into perceptually closed objects that could exist in a Euclidean 3-dimensional world but not be categorized. Findings show processing differences between the matched sets of the intact real and pseudo objects only after 175 ms during the N3 complex, confirming that, as designed, real, and pseudo objects are well-matched for low-level visual feature processing. All drawings were impoverished by deleting random squares of pixels across 8 *fragmentation levels* in a series using the algorithm of Snodgrass et al. (1987). Levels 1 (intact) to 6 (most fragmented) were used here. Such random impoverishment methods have the following advantages. First, fragmentation is not determined by a theory that could bias the features and properties in the stimuli, it does not depend on subjective judgments, and it produces stimuli that are challenging to categorize. Second, the stimuli do not depend upon uncontrolled variations in individual perceptual processing, as when visual input is impoverished by short presentation duration (Snodgrass et al., 1987; Snodgrass and Corwin, 1988a). Third, no masking is used that could limit top-down processes (Di Lollo et al., 2000). Of 260 fragmentation series for real objects, Snodgrass and Corwin (1988a) produced 150, and the first author produced 110 using the same software for a prior study (Schendan and Kutas, 2002). Two hundred of these series were chosen for the behavioral study that accompanied the fMRI version and generated normative data (Ganis et al., 2007) that were then used to choose 128 series, each of which had 2 fragmentation levels (low vs. high) that met two criteria: (1) At least 75% of people named each object correctly at both levels based on naming norms. (2) For each object, response times (RTs) were faster numerically for the low than high fragmentation level. Of these 128, 96 were from the Snodgrass and Corwin (1988a) set. Low fragmentation was intended for the LI condition; high fragmentation was intended for the MI condition. For pseudo objects, the same software fragmented these images to the same level as their corresponding real objects. These methods produced list I and its three orders used for fMRI (Ganis et al., 2007), and, for this ERP version, we added a second list (II): An object (real or pseudo) depicted at a higher fragmentation level in one list was presented instead at a lower fragmentation level in the other list, and *vice versa* (i.e., level 1, 2, 3, 4, 5, or 6 in list I became level 6, 5, 4, 3, 2, or 1 in list II, respectively). Each list was shown in 3 pseudo-random orders of intermixed, real, and pseudo objects counterbalanced across subjects. Based on normative data (Snodgrass and Vanderwart, 1980), stimuli chosen for the MI and LI real object conditions, respectively, did not differ in visual complexity (2.9 vs. 2.9), name agreement (86 vs. 87%), image agreement (3.7 vs. 3.6), familiarity (3.4 vs. 3.2), name frequency (18 vs. 15), and acquisition age (2.6 vs. 2.8).

Pseudo objects served two goals. First, they enable an *impoverished-real-object effect* to be revealed. By design (Schendan et al., 1998), these pseudo-objects match real object versions in low-level features, perceptual properties, and coherent object structure but, unlike real objects, activate knowledge weakly, if at all. Second, they served as catch trials to ensure that people categorized the real objects. Pseudo objects cannot be categorized by design, enabling subjects who do not reliably discriminate real and pseudo objects to be excluded. Catch trials validate the key press reports objectively and independently. While overt naming unambiguously reveals categorization accuracy (Schendan and Maher, 2009), it has the disadvantages of (a) demanding additional lexical retrieval not required for categorization *per se* (Damasio et al., 1996) and (b) introducing movement artifacts. Importantly, key press reports of categorization are reliable (Snodgrass and Yuditsky, 1996), and ERP effects are similar for key press and naming measures of categorization (Schendan and Maher, 2009). The design aimed to equate numbers of categorized and uncategorized trials so as not to discourage people from trying to categorize. While this necessitated using half the number of trials for pseudo relative to real objects, ample trials remained for valid ERPs in all conditions, as confirmed by visual inspection to ensure reliable waveforms from each subject. However, real and pseudo versions therefore also could not be presented in matched yoked pairs, as in our prior work showing no ERP effects before 175 ms (Schendan et al., 1998). Therefore, while, for completeness, the present study assesses ERP type effects before 175 ms, these likely reflect low-level feature differences, not just knowledge. Consequently, we focus conclusions on type effects after 175 ms that replicate those with the fully matched set (Schendan et al., 1998) and any impoverished-real-object effects (i.e., impoverishment by type interaction). Further any such interactions will be interpreted with this caveat in mind.

### Design and procedure

**Figure 2:**
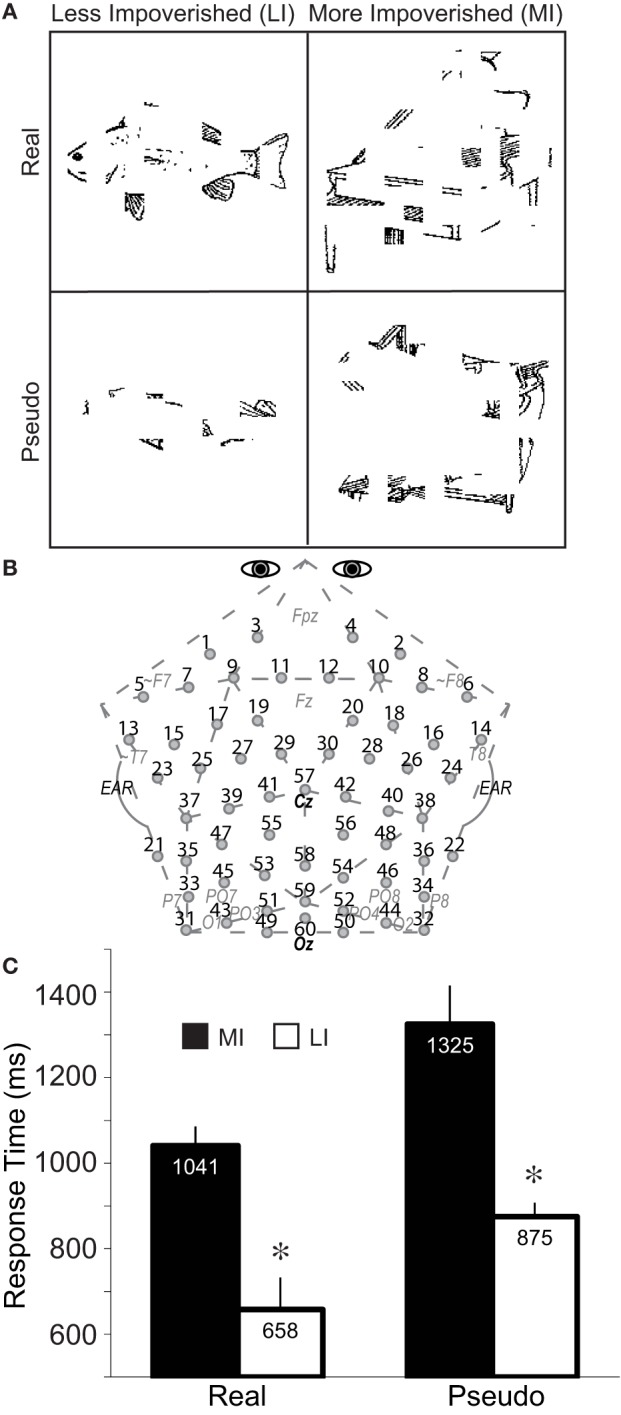
**Method and performance**. **(A)** A 2 × 2 repeated measures design was used with impoverishment (less, more) and object type (real, pseudo) as factors. Fragmented line drawings of real and pseudo objects were shown. Pseudo objects had been created by re-locating the local parts of each real object to create a closed, perceptually coherent but unknown more global shape that could exist in a Euclidean 3-dimensional world but cannot be categorized (Schendan et al., 1998). Subjects pressed “1” to report that they categorized the object or, if not, they pressed “2,” as soon as possible after the picture appeared. A median split of the RTs to real and pseudo objects, separately, for correct responses (i.e., 1 for real objects, 2 for pseudo objects) separated these conditions into more (MI) and less (LI) impoverished conditions. Shown are real objects of an LI fish at fragmentation level 3, and MI piano at level 4, and an LI pseudo-fish at level 5, and MI pseudo-piano at level 4; note, sample stimuli reflect the consistent finding that more fragmented real objects are related to slower RTs, whereas more fragmented pseudo objects are related to faster RTs. Stimuli subtended 6 by 6 degrees of visual angle, on average, with a visual contrast of approximately 30% (dark pixels against a brighter background). **(B)** Custom 60-channel geodesic montage for EEG recording (Electrocap International). Circles show electrode locations. Numbers label each electrode. Approximate locations of 10–20 sites are shown in gray italics; site 57 is at Cz, site 60 is Oz; pairs 31–32, and 49–50 are 1 cm below the inion. **(C)** Response times to MI and LI real and pseudo objects. Error bars show the 95% confidence interval (Morey, 2008). ^*^Significant impoverishment effect.

A 2 × 2 repeated measures factorial design (Figure 2A) included factors of impoverishment (LI, MI) and object type (real, pseudo). General health history and Edinburgh Handedness (Oldfield, 1971) questionnaires were administered before each session. The ERP session started with instructions on the computer screen that subjects paraphrased aloud, and any misconceptions were corrected. They were instructed on the task, to maintain eye gaze on the fixation mark at the center of the screen, and blink only in the fixation period. They then received 10 practice trials using the experiment methods but different stimuli. On each experiment trial, a fixation period of 5400–5700 ms preceded each picture, which was presented for 1000 ms while subjects decided whether they could categorize each object. They pressed “1” as soon as they knew what the object was, or “2” if they did not know, as quickly as possible without sacrificing accuracy. Participants were informed that categorization would be challenging by design because the images were degraded. They were not informed that some objects were impossible to categorize (i.e., pseudo objects) and so, from the subjects' perspective, pseudo objects were just images that they could not categorize (i.e., possible “real” objects that they failed to categorize).

### Electroencephalography (EEG)

The *ERP System* software (Holcomb, 2003) presented stimuli and recorded and analyzed data on PCs running Windows XP. A *Belkin* Nostromo game pad detected responses. EEG data were recorded at 200 Hz (bandpass 0.01 to 100 Hz; SA Instrumentation Company) from 60 Ag/AgCl electrodes attached to a plastic cap (Figure 2B). Cap, nose, and right mastoid electrodes and one below the right eye (monitoring eye blinks) were referenced to the left mastoid. Bilateral eye electrodes (monitoring eye movements) were referenced to each other. Using *ERP System* software and standard methods (Luck, 2005), 27% of EEG trials were excluded from analysis that contained above threshold blinks (determined for each individual participant, and based on polarity inversion between the lower eye and right frontopolar electrode 4), eye and other movement artifacts (based on peak to peak amplitude for the bilateral eye electrodes and individual electrodes, respectively), muscle activity (based on high frequency local peaks within a time period). ERPs were calculated offline by averaging artifact-free EEG in each condition, time-locking to object onset with a 100 ms pre-stimulus baseline, and re-referencing to the mean of both mastoids. To compare with some prior studies, ERPs were also re-referenced to the common average of all electrodes, except bilateral eyes, and plotted positive up, which highlights the resemblance between frontopolar N3 effects with the mastoid reference (e.g., site 3) and occipitotemporal positivity (“P3”) effects with the common average reference (e.g., site 22).

### Analyses

Accuracy and the RTs and ERPs on correct trials were analyzed. “Correct trials” for real objects corresponded to “categorized” responses (i.e., hits). “Correct trials” for pseudo objects corresponded to “not categorized” responses (i.e., correct rejections). For each subject, the RT median for real and pseudo objects, separately, split trials into MI (slower) and LI (faster) conditions, which was the main analysis in the fMRI version and found to be most valid way to subdivide the trials to reveal impoverishment effects (Ganis et al., 2007). For the fMRI version, data were also re-analyzed using fragmentation level to define MI and LI conditions, revealing the same results as for the median RT split, though slightly less significant, consistent with the known performance variability among fragmentation series (Snodgrass and Corwin, 1988a). Consequently, categorization performance (i.e., median RT split), as opposed to fragmentation level, best captures the full set of image characteristics that defines each stimulus' goodness (i.e., impoverishment) for a category decision: Individual RT captures all factors that impoverish each picture and affect the category decision, and the results define the full range of processes that contribute to the visual constancy of object cognition. Thus, for completeness, as for fMRI, data were analyzed in two additional ways: (a) over fragmentation levels and (b) RT median split for only levels 3, 4, and 5 for which average visual complexity was equated between the MI and LI sets. For the latter (b), median RTs were re-computed for levels 3–5 and trials split into MI and LI conditions, accordingly: 98 of 128 real objects in list I; 75 of them in list II (fewer due to the level switch); for correct trials after artifact rejection, about 52 real and 39 pseudo object trials were analyzed from each subject on average. For the former (a), to assess whether results would change if fragmentation defined MI and LI levels, ERP data were re-analyzed using fragmentation level to define MI (levels 4–5) and LI (levels 2–3) conditions; these levels yielded similar trial numbers in each condition, while also minimizing perceptual differences between MI and LI trials. Indeed, as for fMRI, the ERP results defined using fragmentation replicated those using the RT definition (both all trials and levels 3–5). In sum, regardless of how impoverishment is defined, results remained the same. As results of all analyses did not differ, the best controlled analysis that yielded the largest effects (i.e., RTs for levels 3–5) is reported.

Mean ERP amplitudes, time windows and electrodes were chosen based on prior ERP studies of vision and categorization; all components analyzed here have known scalp distributions (Picton et al., 2000; Luck, 2005): (a) From 145 to 160 ms assessed the VPP/N170 (Schendan and Lucia, 2010). (b) The N3 complex is a negative-going ERP over frontal locations that can sometimes invert polarity over occipitotemporal locations between 200 and 700 ms with a peak typically around 350 ms. As the N3 complex has subcomponents that can differ over time, the frontal N3 and its occipitotemporal counterparts were assessed from 200 to 299, 300 to 399, and 400 to 499 ms; note, the 300 to 499 ms times also assessed the centroparietal N400 (Schendan and Maher, 2009). (c) From 500 to 699 ms assessed the P600, (d) 700 to 899 ms assessed the SW, and both these time periods after 500 ms also assessed the LPC. Focal spatiotemporal planned contrast ANOVAs isolated effects (*df* s[1, 18]) to lateral pairs or midline sites and times when an ERP was maximal and overlapped least with others: (a) 145 to 160 ms for the VPP at pair 29–30, and its polarity inverted N170 at occipitotemporal pair 33–34; (b) 200 to 299, 300 to 399, and 400 to 499 ms for frontopolar ERPs at pair 3–4 and occipitotemporal polarity inverted counterparts at pair 21–22, and 300 to 399 and 400 to 499 ms for frontocentral negativities at pair 29–30; (c) pair 47–48 from 300 to 399 and 400 to 499 ms for the centroparietal N400; (e) pair 53–54 from 500 to 699 and 700–899 ms for the parietal P600 and broad LPC; (d) 500 to 699 and 700 and 899 ms for the SW at frontocentral pair 11–12 and broad LPC. The Bonferroni method corrected for planned comparison of multiple sites within a time period by dividing the alpha of 0.05 for each time period by the number of sites tested (**Table 3**).

Mixed ANOVAs included 2 Impoverishment (MI, LI) × 2 object Type (real, pseudo) within-subjects factors and between-subject nuisance variables of list (I, II) and order (A, B, C) of no interest and not reported. For ERP ANOVAs, a within-subjects factor of electrode was added, and midline (labeled as such) and lateral electrodes (unlabeled) were analyzed separately to assess hemispheric asymmetries with an added within-subject factor of hemisphere in lateral ANOVAs, and, in midline ANOVAs, lobe (parietal [sites 57, 58], occipital [59, 60]). The Huynh–Feldt correction was applied for violations of the sphericity assumption. For brevity, only results for critical factors of impoverishment and type, and their interactions are reported, as scalp location effects alone are not of theoretical interest. Degrees of freedom (*df* s) are listed with the first report of each effect. Planned simple effects tests assessed the impoverishment by type interaction for focal results, which target specific ERP components.

### Source estimates

Theoretically, the inverse problem of localizing the cortical sources of electromagnetic data recorded from the scalp has no unique solution. Standardized low resolution brain electromagnetic tomography (s*LORETA*) estimates the sources (Pascual-Marqui, 2002). The sLORETA software computes the three-dimensional (3D) distribution of current density using a standardized, discrete, 3D distributed, linear, minimum norm inverse solution. Localization is data-driven, unbiased (even with noisy data), and exact but has low spatial precision due to smoothing assumptions resulting in highly correlated adjacent cortical volume units. A realistic head model constrains the solution anatomically using the structure of cortical gray matter from the Montreal Neurological Institute (MNI) average of 152 human brains as determined using the probabilistic Talairach atlas. Images plot the exact magnitude of the estimated current density based on the standardized electrical activity in each of 6239 voxels of 5 mm^3^ size. The sLORETA software computed the sources of the grand average ERPs over all sites, except nose, and eyes (Pascual-Marqui, 2002). Electrode coordinates were digitized using an infrared digitization system, and imported into *LORETA-Key* software. This coordinate file was then converted using the sLORETA electrode coordinate conversion tools. The transformation matrix was calculated with a regularization parameter (smoothness) corresponding to a signal-to-noise ratio of 50. We localized the difference waves of each of the 4 effects (**Figure 7**). The ERP difference data are akin to the signal differences between fMRI conditions and so limit sources to those that could reflect fMRI activation, and difference waves may reveal weaker sources better (Luck, 2005).

### Subjects

Ethical approval granted through the Institutional Review Board of Tufts University. Participants were 39 healthy Tufts University students or people from the greater Boston community. 1 person was excluded due to a data recording error and another due to strabismus. Data were analyzed from 24 of the 37 subjects remaining who met the following inclusion criteria: (a) The *d*′-value was 1.0 or better (μ = 2.35) based on the hit rate for real objects, and false alarm rate to pseudo objects out of the total trials eliciting a response (i.e., excluding ambiguous no responses). (b) Two-thirds or more of real and pseudo object trials were correct to ensure valid RTs and ERPs following artifact rejection. (c) Visual inspection of each subject and condition confirmed each ERP was valid (μ = 28 and 26 trials, respectively, at levels 3–5) (Picton et al., 2000). The analyzed group was half female, aged μ = 21.2 years (range 18.0–29.8), had education μ = 14.4 years (range 12–20), and handedness score μ = 97.8 (right-handed).

## Results

### Performance

Performance replicated the fMRI version (Ganis et al., 2007). Results of signal detection theory (SDT) analyses with logistic distributions (Snodgrass and Corwin, 1988b) validated category decision accuracy. Subjects reliably decided that real objects were categorized and pseudo objects were not. The average discrimination index ($d_{\text{L}}^{\prime }$) was 4.13 (corrected rates: 73.6% hits, 6.9% false alarms), demonstrating very high detection of knowledge conveyed by real objects. The average criterion (*C*_L_) was 0.97, which was above the neutral 0 level [*t*_(23)_ = 7.80, *p* < 0.001], indicating subjects were slightly biased to be conservative in reporting detection of knowledge. Subjective probability that each picture could be categorized can affect ERPs, such as P300-like potentials (e.g., P600, LPC) (Johnson, 1986), so, to assess this, response rates were computed collapsed across both object types (real, pseudo). Results showed that subjects decided that they could categorize about half of the pictures: 50.0% categorized vs. 49.0% uncategorized [levels 3–5, *F*_(1, 18)_ = 0.13, *p* = 0.72]. This 50:50 decision rate demonstrates that subjective probability of response type (and picture categorizability) cannot explain ERP effects.

RTs (Figure 2C) were faster in LI than MI conditions, by design, *F*_(1, 18)_ = 182.83, and for real than pseudo objects, *F*_(1, 18)_ = 25.14 (*p*s < 0.0001). LI were faster than MI, but more so for pseudo than real objects, resulting in an Impoverishment by type interaction, *F*_(1, 18)_ = 9.25, *p* = 0.007. Since this could be due to the overall slower RTs for pseudo than real objects, normalized RT scores (MI-LI/MI) were analyzed, demonstrating that impoverishment effects were actually greater for real (score = 0.36) than pseudo objects (score = 0.33), *F*_(1, 18)_ = 6.09, *p* = 0.024. Results do not reflect speed-accuracy trade-offs, because RTs and accuracy for real objects did not correlate across subjects (*r* = 0.14, *p* > 0.5). Analyses of the relation between fragmentation level and RT confirmed that, as designed, RT correlated with fragmentation level for real objects, *r* = 0.61, *p* < 0.001.

### ERPs

**Figure 3:**
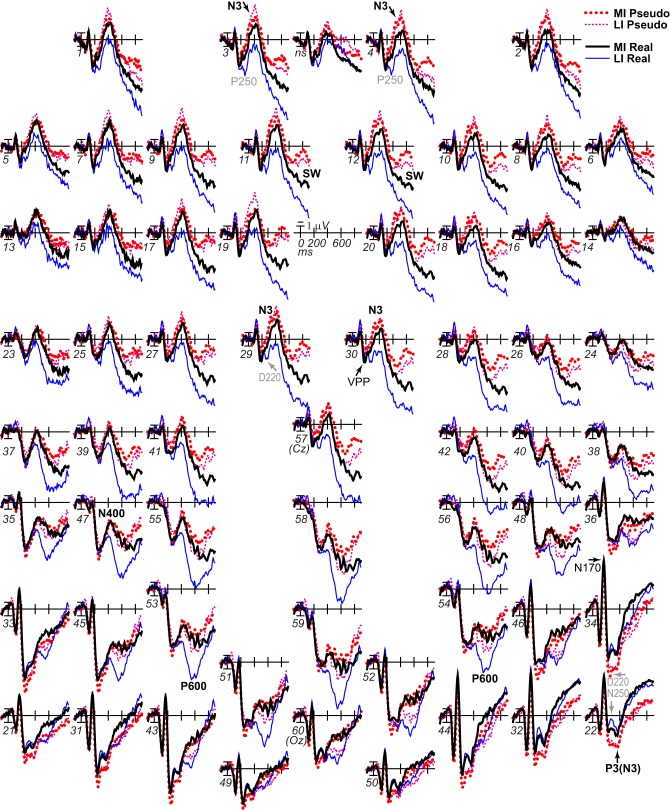
**ERP effects of impoverishment and object type**. Grand average ERPs at all channels show effects of impoverishment (more [MI], less [LI] impoverished) and object type (real, pseudo). Unless otherwise specified, ERPs in this and following figures were low-pass filtered at 30 Hz and were referenced to the average of left and right mastoids. Numerals label electrode locations; ns, nose. Impoverishment and object type modulated the N3 complex (including P250/N250 and D220 components; components inverted polarity between frontal and occipitotemporal sites), N400, P600, and slow wave (SW) components after 200 ms, but not the earlier VPP/N170.

**Figure 4:**
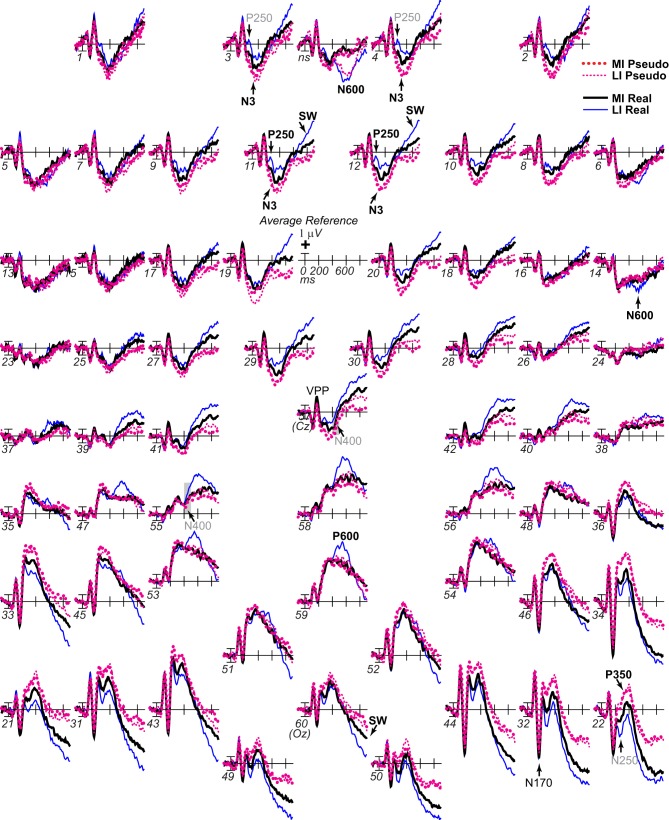
**ERP effects of impoverishment and object type with common average reference**. As in Figure 3, grand average ERPs are plotted at all sites, but, in order to compare with other work, the reference was computed using the average of all scalp sites (i.e., the “average reference”), and ERPs were instead plotted positive up. ERP effects of impoverishment (more [MI], less [LI] impoverished) and object type (real, pseudo) are shown. Compared with Figure 3, with the average reference, here, the parietal P600 inverts polarity over lateral frontal and frontopolar sites to an N600, especially at the right. The late SW from 500 to 900 ms has an occipital distribution that inverts polarity over frontocentral sites near the midline, and is larger over the left hemisphere. Note, with the common average reference, the N400 pattern (gray shadow) cannot be discerned from the overlapping N3 and P600 times, highlighting the importance of using the same reference sites across studies to identify components and draw conclusions; studies analyzing data using the common average reference may misattribute N3 and/or P600 effects to the N400.

The aim was to determine when impoverishment and object type interact such that the impoverishment effect is larger for real than pseudo objects. Table 1 summarizes ERP results, which were most consistent with late parietal-prefrontal PHT, MUSI, and decision theories. After 200 ms, impoverishment affected knowledge activation, modulating the N3 complex, N400, P600, and SW (Figures 3, 4); note, as results suggested no distinct LPC effects, henceforth, we refer only to the P600 and the SW.

#### N170/VPP

From 145 to 160 ms, omnibus results showed that object type interacted significantly with lateral and midline electrode sites (Table 2). Focal spatiotemporal analyses showed a marginal type effect at frontocentral pair 29–30 (Table 3) where positivity was slightly greater for real than pseudo objects.

**Table 2:** ***F–*values for significant effects in omnibus lateral (Lat) and midline (Mid) ANOVAs with impoverishment and object type factors at each time period after 200 ms**.

**ERP**	**VPP/N 170**		**N3**				**N3 and N400**		**P600**		**SW**	
**Time (ms)**	**145–160**		**200–300**		**300–400**		**400–500**		**500–700**		**700–900**	
**Source**	**Lat**	**Mid**	**Lat**	**Mid**	**Lat**	**Mid**	**Lat**	**Mid**	**Lat**	**Mid**	**Lat**	**Mid**
Impoverishment (I)	–	–	–	–	–	–	15.0^**^	19.8^**^	38.1^**^	49.7^**^	11.9^**^	–
Type (T)	–	–	–	–	6.3^*^	4.4^*^	14.2^**^	17.8^**^	23.0^**^	30.0^**^	112.3^**^	46.8^**^
I × Hemisphere (H)	–	–	–		–		–		–		5.8^*^	
I × Electrode (E)	–	–	5.1^**^	–	–	–	2.8^*^	–	4.8^**^	–	5.2^*^	13.2^**^
I × Lobe (L)	–	–		–		–		–		–		16.1^**^
I × L × E	–	–		–		–		7.4^*^		14.3^**^		7.0^*^
T × E	4.57^**^	8.03^*^	35.7^**^	28.4^**^	48.6^**^	52.7^**^	22.3^**^	20.9^**^	32.8^**^	32.5^**^	54.4^**^	81.3^**^
T × E × H	–	–	3.0^**^		2.5^**^		3.4^**^		4.2^**^		2.1^*^	
T × L	–	10.2^**^		40.1^**^		45.4^**^		14.2^**^		50.0^**^		81.9^**^
T × L × E	–	–		–		–		11.1^**^		39.8^*^		5.7^*^
I × T	–	–	10.6^**^	–	5.5^*^	–	9.1^**^	5.3^*^	5.4^*^	5.1^*^	–	–
I × T × E	–	–	–	–	–	–	–	–	–	5.1^*^	–	7.6^*^
I × T × L × E	–	–		–		–		5.4^*^		8.6^**^		–

df (1, 18) for lateral effects of I, T, I × T, I × H, and all Midline effects and their interactions; df (27, 486) for Lateral Electrode effects and their interactions. Times rounded to nearest 5 ms. Epsilon values for lateral sites at each time period (start-end time in ms): 145–160 (T × E = 0.132), 200–300 (T × E = 0.16; I × E = 0.16; T × E × H = 0.27), 300–400 (T × E = 0.17; T × E × H = 0.35), 400–500 (T × E = 0.20; I × E = 0.16; T × E × H = 0.22), 500–700(T × E = 0.21; I × E = 0.12; T × E × H = 0.25), 700–900(T × E = 0.15; I × E = 0.10; I × T × E = 0.12; T × E × H = 0.32). −p > 0.05;

* p < 0.05;

** *p < 0.01*.

**Table 3:** ***F*–values for significant effects of improvements (I) and type (T) in focal ANOVAs at specific lateral electrode pairs and times**.

**ERP**	**VPP**	**N3 complex**									**N400**	**P600**	**SW**	
**Site**	**FC 29–30**	**Fp 3–4**			**FC 29–30**			**OT 21–22**			**CP 47–48**	**P 53–54**	**FC 11–12**	
**Time (ms)**	**145–160**	**200–300**	**300–400**	**400–500**	**200–300**	**300–400**	**400–500**	**200–300**	**300–400**	**400–500**	**400–500**	**500–700**	**500–700**	**700–900**
**FOCAL OMNIBUS**														
T	4.6^+^	44.52^**^	90.5^**^	48.52^**^	14.92^**^	22.88^**^	23.22^**^	26.76^**^	46.99^**^	20.8^**^	5.54^+^	30.65^**^	24.1^**^	141.8^**^
I	–	7.21 b^*^	14.12^**^	9.11^**^	–	6.13^+^	11.56^**^	7.7 b^*^	–	–	15.05^**^	47.26^**^	8.1a^*^	12.91^**^
T × H	–	–	–	–	–	–	–	–	–	–	–	4.95^+^	–	–
I × T	–	5.88^+^	9.31^**^	5.55^+^	5.78^+^	–	7.12^+^	–	–	–	6.78^+^	5.78^+^	–	4.9^+^
I × T × H	–	–	–	–	–	–	–	4.47^+^	–	–	–	–	–	5.44^+^
**FOCAL PAIRWISE CONTRASTS**														
*Real*														
I	–	12.76^**^	21.28^**^	16.66^**^	–	9.96^**^	15.46^**^	4.55^+^	–	–	17.07^**^	33.65^**^	10.3^**^	21.87^**^
*Pseudo*														
I	–	–	–	–	–	–	–	–	–	–	–	11.67^**^	–	–
I × H	–	–	–	–	–	–	–	–	–	–	–	–	5.86^+^	–
*Less Improvished (LI)*														
T	–	26.86^**^	45.88^**^	27.58^**^	16.68^**^	18.65^**^	18.52^**^	11.44^**^	20.55^**^	9.06^**^	8.85^**^	37.33^**^	14.1^**^	78.14^**^
T × H	–	–	–	–	–	–	–	5.46^+^	–	–	–	–	–	–
*More Improvished (MI)*														
T	–	17.03^**^	15.17^**^	–	4.8^+^	4.73^+^	7.27^+^	37.88^**^	39.8^**^	24.27^**^	–	–	8.98^**^	55.92^**^

Hemisphere (H). df(l, 18). Times rounded to nearest 5 ms. Fp, Frontopolar; FC, Frontocentral; OT, Occipitotemporal; CP, Centroparieta1; P, Parietal. –p > 0.05;

+ p < 0.05;

** p < 0.01; correction for multiple comparisons: alpha was 0.025 for 145–160 ms at pairs 29–30 and 33–34 and 500–700 ms at pairs 53–54 and 11–12(a^*^ p < 0.0250); alpha was 0.0167 for 200–300 and 300–400 ms at pairs 3–4, 29–30, and 21–22 (b^*^ p < 0.0167); alpha was 0.0125 for 400–500 ms at pairs 3–4, 29–30, 21–22, and 47–48(c^*^ p <.0125).

#### N3 complex and N400

Omnibus results at N3 and N400 times from 200 to 500 ms (Table 2) showed significant effects of type and impoverishment. Most important, impoverishment by type interactions were significant at lateral sites the entire time from 200 to 500 ms and at the midline from 400 to 500 ms.

##### N3 complex (200–500 ms)

**Figure 5:**
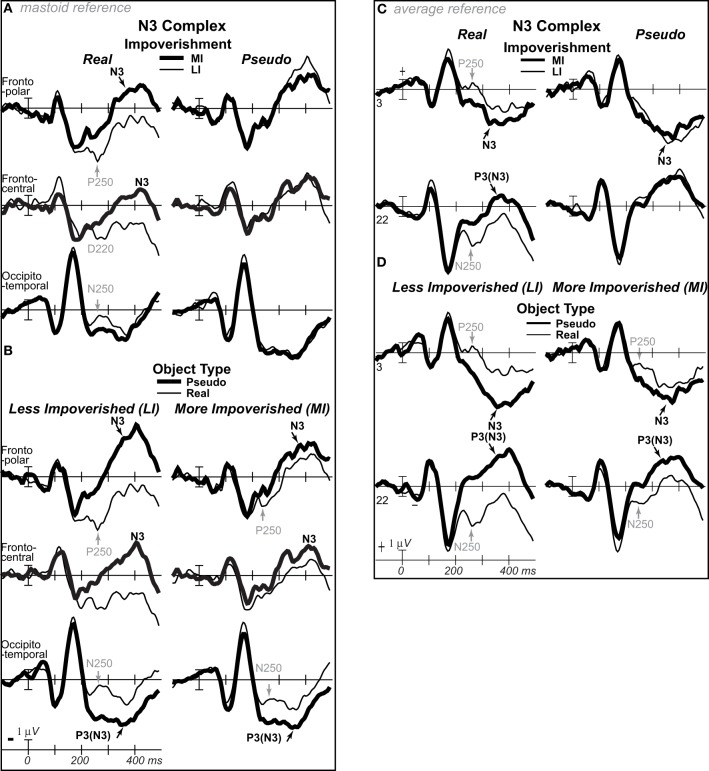
**ERP effects of impoverishment and object type on the N3 complex**. **(A,B)** Shown are sites of the N3 complex maxima (left frontopolar site 3, right frontocentral site 30, right occipitotemporal site 22). Frontal effects inverted polarity to positivity at occipitotemporal sites, especially on the right (“P3[N3]” maximal at site 22), including an N250; note, a D220 index of task difficulty for decisions also inverted polarity between frontocentral and occipitotemporal sites. **(A)** N3 effects of impoverishment shown for real objects and pseudo objects. The frontal N3 showed an impoverished-real-object effect, including a frontopolar P250 component: The frontal N3 components were more negative for MI than LI real objects but not pseudo objects; note, the N3 showed no such effect for pseudo objects, but, in contrast, briefly at the peak, the N3 was instead slightly more negative for LI than MI pseudo objects. The occipitotemporal N250 but not later posterior N3 counterparts showed impoverishments effects for real objects. **(B)** N3 effects of object type shown on LI and MI trials. The N3 complex was larger for real than pseudo objects, and this type effect was larger on LI than MI trials. **(C,D)** To compare with other publications, the reference was computed using the average of all scalp sites (i.e., “common average reference”), and ERPs were plotted positive up. Shown are left frontopolar site 3 and occipitotemporal site 22. **(C)** N3 effects of impoverishment shown, for real and pseudo objects. **(D)** N3 effects of object type shown on LI and MI trials. Here, with the average reference, the effects over occipitotemporal sites become larger than when the bilateral mastoid reference is used instead (see **A,B**): Notice the similarity of effects between frontopolar site 3 in **(A,B)** and occipitotemporal site 22 here [also site 22 in **(A,B)** is more like site 3 here]. Crucially, the frontopolar ERPs with a mastoid reference [e.g., P250, N3 in **(A,B)**] correspond, with the average reference shown here, to the occipitotemporal ERPs (e.g., N250, P3(N3) at site 22 here). This demonstrated a clear link between the present and prior research on the frontocentral N3 complex and its subcomponents, and prior research on the occipitotemporal N250 and Ncl, which were defined using the nose or average reference, as shown here; note scalp distribution shapes with nose and average reference are similar. Like the frontopolar P250/N3 with the mastoid reference (see **A,B**), here with an average reference, the occipitotemporal N250 and P3(N3) show the impoverished-real-object effect, being more positive for MI than LI real objects but not pseudo objects, and this effect inverts polarity over frontopolar sites to P250 and N3 effects. Further, like the frontopolar P250 and N3 with the mastoid reference (see **A,B**), here with an average reference, the occipitotemporal N250 and P3(N3) show object type effects, being more positive for pseudo than real objects on LI and MI trials, and these effects invert polarity over frontopolar sites. The whole head ERPs in Figure 4 demonstrate that this polarity inversion of effects occurs between frontal sites toward the midline (3–4, 11–12, 19–20, 29–30) and more lateral occipitotemporal sites with a right hemisphere maximum (22, 32, 34), especially for the N250, consistent with the known right lateralization of the N250 (i.e., N250r).

Focal spatiotemporal results demonstrated that the frontal N3 was more negative for (a) MI than LI stimuli for real objects only (Figures 3, 5A) and (b) pseudo than real objects on LI more than MI trials (Figures 3, 5B). Occipitotemporal counterparts showed the same but with opposite polarity (i.e., more positive). Specifically, the results (Table 3) showed main effects of type were significant the entire time from 200 to 500 ms at frontopolar, frontocentral, and occipitotemporal sites. Main effects of impoverishment were significant at frontopolar sites the entire time, frontocentral sites from 400 to 500 ms, and occipitotemporal sites from 200 to 300 ms. The critical impoverishment by type interactions were significant at frontopolar sites from 300 to 400 ms; note, interactions were marginal at other times frontally and occipitotemporally from 200 to 300 ms. Planned contrasts (Table 3) showed that only real objects had significant impoverishment effects during the entire frontopolar N3 (200 to 500 ms) and later frontocentral N3 (300 to 500 ms); note, this effect was marginal on the occipitotemporal N250 from 200 to 300 ms. Further, type effects were significant, for LI, at all times and N3 sites and, for MI, from 200 to 400 ms at frontopolar sites and all times at occipitotemporal sites; note, for MI, type was marginal at frontocentral sites. With a common average reference, N3 effects split about evenly between frontal and occipitotemporal sites (Figures 4, 5C,D).

##### N400 (300–500 ms)

**Figure 6:**
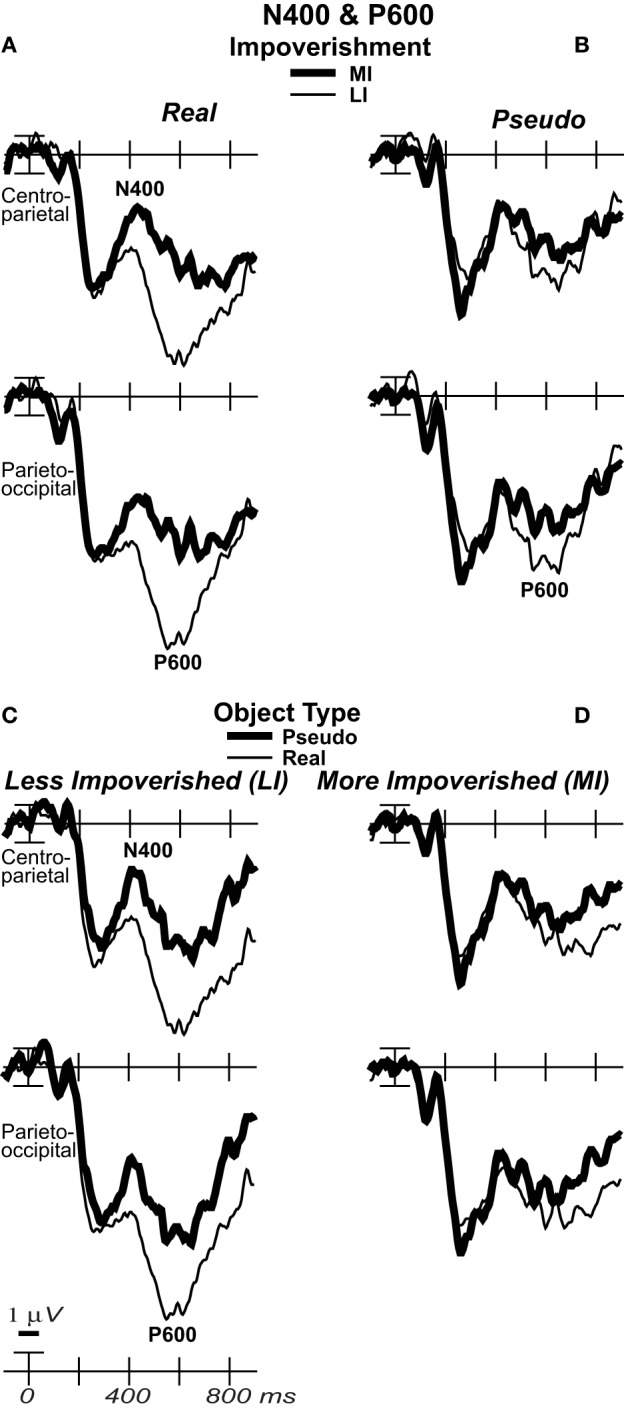
**Effects of impoverishment and object type on the N400 and P600**. Grand average ERPs at focal sites of the centroparietal N400 and parietal P600 plotted negative up. N400 and P600 impoverishment effects shown for **(A)** real objects and **(B)** pseudo objects. **(C)** N400 and P600 object type effects shown on LI and **(D)** MI trials, which showed no type effect. From 400 to 700 ms, impoverished-real-object effects were found on the N400 and P600. Positivity was greater on LI than MI trials, and this impoverishment effect was larger for real than pseudo objects, which showed no such effect on the N400. The P600 was the first ERP to show impoverishment effects for both real and pseudo objects and in the same direction.

Focal results demonstrated that the N400 was less negative for LI real objects than all other stimuli, demonstrating impoverished-real-object effects (Figures 3, 4, 6). Specifically, the results (Table 3) showed significant impoverishment effects at centroparietal pair 47–48 from 400 to 500 ms, though type effects and the impoverishment by type interaction were marginal. Planned contrasts (Table 3) supported the critical interaction, as impoverishment was significant for real objects only, and type was significant for LI stimuli only. Notably, while the earlier frontal N3 showed type effects for both MI and LI stimuli, type effects between 400 and 700 ms at the parietal N400 and P600 sites, occurred only for LI objects, dissociating the frontal and parietal ERPs.

#### P600/LPC (500–700 ms)

Around 500 ms, N3 complex effects ended, and the parietal P600 showed impoverished-real-object effects, as the impoverishment effect was larger for real than pseudo objects. Positivity was greater for LI than MI stimuli and for real than pseudo objects, and the impoverishment effect was larger for real than pseudo objects (Figures 3, 6). With a common average reference, a left mid-parietal P600 inverted polarity to an N600 at right frontal sites (Figure 4). Accordingly, omnibus results from 500 to 700 ms resembled those from 400 to 500 ms, demonstrating type and impoverishment effects and their interaction (Table 2).

Focal results (Table 3) at parietal pair 53–54 showed significant effects of impoverishment and type, though their interaction was marginal. Planned contrasts (Table 3) showed impoverishment was significant for both object types for the first time between 500 and 700 ms, as earlier ERPs showed impoverishment effects only for real objects. Further, type was significant for LI stimuli only. These results confirm the impoverished-real-object effect on the P600 and dissociate it from other ERPs.

#### SW/LPC (500–900 ms)

Around 700 ms, positivity on a broad anterior SW was greater for LI real objects than MI ones, which was greater than for LI pseudo objects than MI ones, and type effects continued (Figure 3). With a common average reference, the SW was a negativity at occipital sites that inverted polarity to positivity over mid-frontal sites (Figure 4). Omnibus results from 700 to 900 ms (Table 2) showed impoverishment and type effects continued, but the impoverished-real-object effect was only at the midline where the impoverishment by type by electrode interaction was significant due to impoverishment effects for real but not pseudo objects at central more than posterior midline sites.

Focal results at frontocentral pair 11–12 (Table 3) showed effects of type and impoverishment from 500 to 900 ms, and impoverishment and type interacted marginally from 700 to 900 ms. Planned contrasts (Table 3) showed impoverishment was significant for real objects from 500 to 900 ms and marginal for pseudo objects from 500 to 700 ms (LPC time only). Further, unlike the N400 and P600, the N3 and SW showed type effects for both LI and MI stimuli. Thus, no distinct LPC effects were observed, and the anterior SW from 700 to 900 ms showed impoverishment effects for real objects only.

### N3 onset

To define precisely when the impoverished-real-object effect starts, the onset of N3 effects was defined as the time when 15 consecutive points first become significant in a series of point-by-point *F*-tests (Picton et al., 2000) at focal frontopolar pair 3–4 and right occipitotemporal site 22, as frontal N3 effects were bilateral and occipitotemporal N250 effects were larger on the right. The criterion was met for the onset of type effects with LI stimuli by 230 ms. However, omnibus and focal results confirmed type and impoverishment effects during the N3 so it is informative to consider fewer consecutive times. The results thereby also suggested an onset around 250 ms for the impoverished-real-object effect when the most consecutive significant points showing this interaction were at frontopolar site 3 (7 points, *p*s < 0.05, plus 1, *p* = 0.084). Simple effects tests defined the start of impoverishment effects for real objects likewise as 255 ms at frontopolar site 4 (site 3 onset at 245 ms, 13 points, *p*s < 0.05, plus 2, *p*s < 0.064). Type effects started around the same time posteriorly regardless of impoverishment but ~50 ms later on the frontopolar N3 for MI relative to LI stimuli: It started for LI stimuli between 230 and 250 ms (all sites) and, for MI stimuli, from 215 to 220 ms at occipitotemporal site 22 and later at 270 ms at frontopolar site 4 (14 consecutive points) and 280 ms at frontopolar site 3 (7 points, *p*s < 0.019, plus 1, *p* = 0.051). Altogether, these onsets suggest that impoverishment starts to modulate knowledge around the time when knowledge starts to contribute to the category decision: ~250 ms.

### Cortical sources

**Figure 7:**
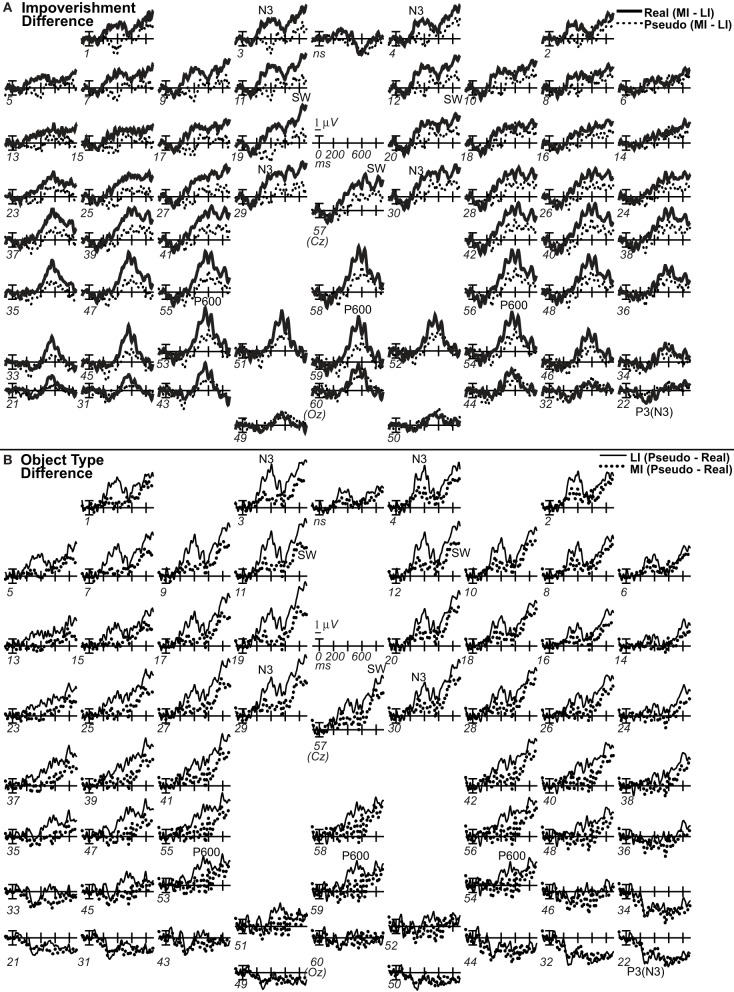
**Grand average difference ERPs computed by subtracting ERPs in two conditions**. For display, waves were low pass filtered at 20 Hz. **(A)** *Difference waves of impoverishment effects*. Effects of impoverishment shown by subtracting the less impoverished (LI) condition from the more impoverished condition (MI). Up is negativity in MI greater than LI. Note, where the impoverishment difference wave was greater for real than pseudo objects reveals the impoverished-real-object effect. **(B)** *Difference waves of object type effects*. Effects of object knowledge shown by subtracting the real object condition from the pseudo object condition. Up is negativity for pseudo greater than real objects.

**Figure 8:**
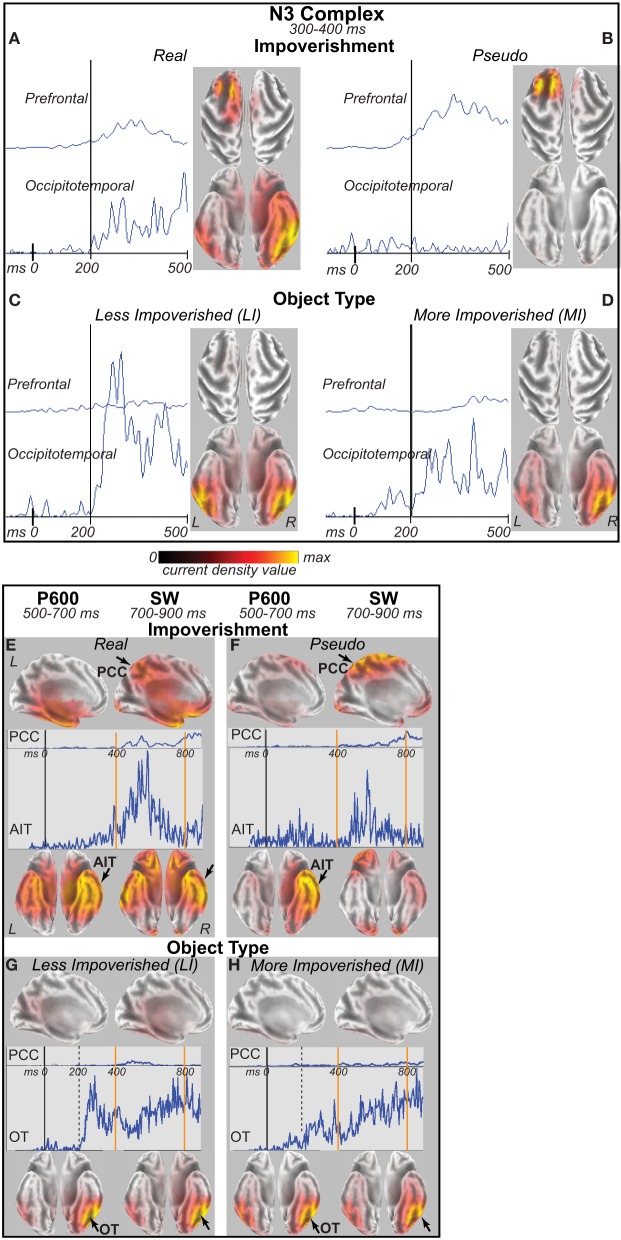
**The sLORETA maps show estimated sources of the difference waves (Figure 7) between two conditions (impoverishment = MI minus LI; type = pseudo minus real) in the grand average ERPs**. Maps shown superimposed on an inflated, canonical MNI152 (Colin) brain. Dark areas are sulci; light areas are gyri. L, left hemisphere; R, right hemisphere. Each brain shows standardized cortical current density distributions, and source activity reflects the location of differential source activity between conditions but not the direction of effects. Scale uses hot colors (red, yellow) for maximal current density value differences. **(A–D)** *N3 Sources*. sLORETA maps shown for the N3 from 300 to 400 ms on dorsal (top) and ventral (bottom) cortical surfaces. Estimated intracranial ERPs plotted on the left for prefrontal (MNI *x y z* coordinates −15 20 65) and occipitotemporal sources (55 −45 –25) between −100 and 500 ms. **(A)** *N3 impoverishment sources for real objects*. Occipitotemporal sources: inferior (BA 20, 60 −40 −20; BA 37, 55 −45 −25) and middle temporal (BA 21, 65 −35 −15; BA 20, 55 −40 −15), fusiform (BA 37, 50 −50 −25; BA 20, 55 −35 −25; BA 19, 45 −70 −20; BA 36, 45 −40 −25), middle occipital (BA 19, 50 −70 −15), lingual (BA 18, 15 −85 −20), and parahippocampal (BA 36, 40 −30 −25) gyri. Prefrontal sources: superior (BA 6, −15 20 65; BA 8, −25 30 55), middle (BA 6, −25 20 60; BA 9, −35 40 40), and inferior frontal (BA 47, 20 25 −20) gyri. **(B)** *N3 impoverishment sources for pseudo objects*. Same prefrontal sources as for real objects. **(C)** *N3 object type sources for LI*. Occipitotemporal sources: fusiform (BA 37, 55 −60 −20, −50 −60 −25; BA 36, 45 −40 −30; BA 19, −50 −70 −20), inferior temporal (BA 20, 50 −55 −20; −60 −55 −20), middle temporal (BA 37, 55 −55 −15, −55 −65 −15; BA 21, 65 −50 −10), middle occipital (BA 37, 50 −65 −15, −50 −65 −15; BA 19, 50 −75 −15), parahippocampal (BA 19, 35 −45 −10) gyri. **(D)** *N3 object type sources for MI*. Same occipitotemporal sources as for LI. **(E–H)** *P600 and slow wave (SW) Sources*. sLORETA maps shown for left medial (top) and ventral (bottom) cortical surfaces. OT, occipitotemporal cortex; AIT, anterior inferior temporal cortex; PCC, posterior cingulate cortex, including precuneus and cuneus. Estimated intracranial ERPs plotted for the voxel showing maximum impoverished-real-object effects from 300 to 400 ms (same as later) in OT (55, −45, −25), 500 to 700 ms in AIT (25, 0 −45), and 700 to 900 ms in PCC (0 −55, 65). **(E)** *Late impoverishment sources for real objects*. AIT sources (maximum BA 20, 25–30 −5 −45) occurred from 450 to 700 ms when the P600 peaks: middle (BA 21, 65 −30 −20) and inferior temporal (BA 20, 60 −35 −20), fusiform (BA 20, 55 −35 −25), parahippocampal (BA 36, 35 −25 −30; BA 35, 30 −25 −25), and other limbic structures (BA 20, 25 0 −45; BA 38, 25 5 −45; BA 36, 25 −5 −40; BA 28, 25 −10 −35). From 500 to 700 ms, limbic lobe dominated (BA 20/38, 25 0 −45), including parahippocampal gyrus (BA 35, 25 −15, −30). From 700 to 900 ms, impoverishment effects in anterotemporal cortex continued and appeared in medial posterior cortex around cingulate (BA 25, 0 5 −10; BA 31, −10 −45 40), cuneus (BA 17, 5 −100 −5), and precuneus (BA 7, 5 −60 65; −5 −50 50), and occipital extrastriate regions (BA 18, 0 −95 −15). The SW effect in PCC is active after 700 ms. **(F)** *Late impoverishment sources for pseudo objects*. P600-like wave in AIT and SW in PCC shown. **(G)** *Late object type sources for LI*, and **(H)** *for* MI: Occipitotemporal cortex only.

For the four difference waves (Figure 7), cortical sources were estimated. The main focus was the time of the N3 peak from 300 to 400 ms (Figures 8A–D). Sources of this impoverishment effect (MI vs. LI) for real objects localized to occipitotemporal and lateral prefrontal areas found with fMRI (Ganis et al., 2007), whereas, for pseudo objects, impoverishment differences localized only to prefrontal areas. Sources of the object type effects (real vs. pseudo) on both LI and MI trials were in occipitotemporal areas. Sources at other times were also estimated. At all times after 200 ms, type effects continued in the same occipitotemporal areas (Figures 8C,D,G,H). Impoverishment sources varied over time and with object type (Figures 8A,B,E,F). The 200 to 300 ms time during the P250/N250 component showed the same impoverished-real-object pattern of sources as the peak N3 time period. Later, from 400 to 500 ms when the N3 ends and the N400 peaks, impoverishment effects for real objects showed only the occipitotemporal source (see intracranial ERP in Figure 8A). Around 450 ms, the maximum source shifted to anterotemporal cortex for both real and pseudo objects, suggesting an additional contribution from this region to the N400. From 500 to 700 ms, the estimated intracranial ERP for the anterotemporal source resembled the scalp P600 impoverishment waveform, which is maximal at this time, and more mediotemporal sources also contributed (Figures 8E,F). From 700 to 900 ms when the late SW dominates, anterotemporal impoverishment activity continued only for real objects. In addition, for both object types, impoverishment effects now appeared in the posterior cingulate cortex (PCC; Figures 8E,F).

### Later ERPs related to RTs

**Figure 9:**
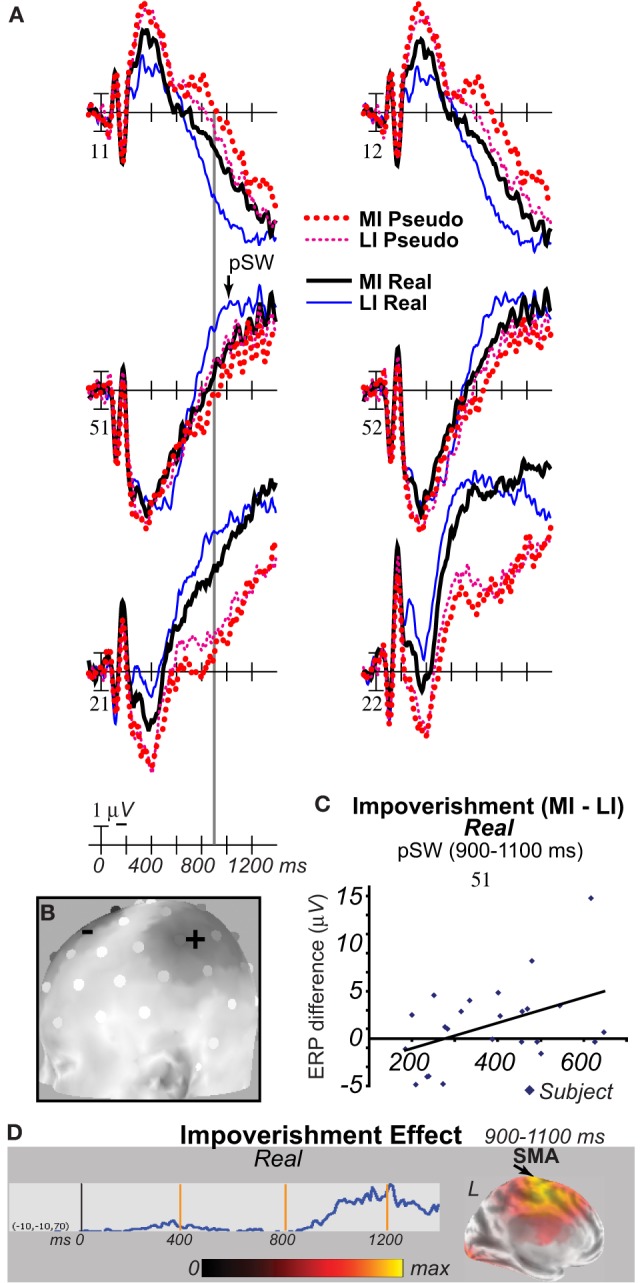
**Late ERP slow waves (SW) show main effects of impoverishment and object type after 900 ms until response, localize to supplementary motor area (SMA), and correlate with RT effects**. **(A)** Grand average ERPs show effects of impoverishment (more [MI], less [LI] impoverished) and object type. ERPs at lateral sites of the SW (11–12), a posterior slow wave (pSW; 51–52), and type effects (21–22) are plotted negative up. Image type and impoverishment modulated distinct ERPs even after 900 and until the latest responses around 1400 ms for MI pseudo objects. An impoverished-real-object effect on a late pSW started after 900 ms (gray line). **(B)** Voltmap generated using sLORETA (default left mastoid reference) shows the distribution of voltage differences over the left hemisphere from 1100 to 1400 ms when only the SMA effect occurs; the distribution is similar from 900 to 1100 ms. Electrodes symbolized by half spheres. The + sites are where the pSW effect is strongest (MI - LI), whereas—sites are the location of the SW over frontal scalp (LI - MI). **(C)** Across subjects, the RT difference correlated significantly with the late pSW effect. Each diamond plots the RT and ERP values for each subject. RT difference on x-axis. ERP amplitude difference on y-axis. The computed linear regression line (solid) is shown. Impoverishment difference for real objects (MI minus LI) from 900 to 1100 ms at site 51 correlated such that, on MI relative to LI trials, as the pSW became more positive, RTs got slower. **(D)** Maps from sLORETA for 900 to 1100 ms on the left (L) medial surface show the late SMA (BA 6, −15 −10 55; 5 −5 65) impoverishment effect for real objects, extending into anterior cingulate gyrus (BA 24, −15 −10 50), and estimated intracranial ERPs show the SMA effect started after 900 ms. Specifically, from 900 to 1100 ms, sources of impoverishment effects for real objects continued in striate/extrastriate and anterior temporal cortex, and, for the first time, were located in left more than right SMA and anterior cingulate, and this effect appeared to correspond to the pSW. From 1100 to 1400 ms, the SMA effect continued, but extended dorsally into superior frontal gyrus (BA 6, −10 [−10 or −15] 70), and posterior effects were minimal or none. At these times, the impoverishment effect for pseudo objects localized to striate/extrastriate areas (BA 17/18) with weaker sources in temporal pole (BA 38, −40 20 −35) and inferior frontal gyrus (BA 47, −50 45 −10). Note, the sLORETA map shows estimated sources of the difference wave (MI - LI) in the grand average ERPs superimposed on an inflated, canonical MNI152 brain (Colin); dark areas represent sulci; light areas represent gyri. The depicted brain shows standardized cortical current density distributions, and source activity reflects the location of differential source activity between conditions but not the direction of effects. Scale shows yellow represents maximal current density value differences. Estimated intracranial ERPs from −100 to 1400 ms were extracted from the voxel showing maximum impoverished-real-object effects at MNI coordinates from 900 to 1400 ms in SMA (−10 −10 70). Solid tics mark the 0 ms stimulus onset and 400 ms intervals post-stimulus.

For completeness and because RTs occurred after the SW, cortical dynamics closer to the motor response were also assessed. EEG was re-analyzed to reject artifacts both between 900 and 1400 ms post-stimulus and during a pre-stimulus baseline of −100 to 100 ms. Analysis times from 900 to 1099 ms captured most MI real object RTs, and 1100 to 1400 ms captured most MI pseudo object RTs. Results showed anterior SW effects of impoverishment continued until 1099 ms and type until 1400 ms. Greater positivity was also found on a left mid-occipital-parietal slow wave (pSW) for MI than LI real objects from 900 to 1400 ms, which inverted polarity anteriorly, and the pSW showed type effects for MI trials until 1099 ms (Figures 9A,B). Critically, no impoverishment by type interactions were found after 900 ms. Both times showed main effects of type and impoverishment laterally, and type at midline sites (*F*s > 10.70, *p*s < 0.005), and type and impoverishment each interacted with lateral electrode (*F*s > 4.33), type with midline electrode and with lobe (*F*s > 29.33), and impoverishment with midline electrode by lobe (*F*s > 28.76), *p*s < 0.003. From 900 to 1099 ms, results also showed interactions of impoverishment by hemisphere, by midline electrode, by lobe (*F*s > 5.4), by electrode by hemisphere (*F* = 2.19), *p*s < 0.04, and by Type by midline electrode (*F* = 9.74, *p* = 0.006). Focal simple effects tests on frontal SW pair 11–12 showed all impoverishment and type effects were significant from 900 to 1099 ms and both type effects from 1100 to 1400 ms (*F*s > 4.51, *p*s < 0.05). Parietal pair 51–52, where the pSW was large, showed impoverishment by hemisphere for real objects from 900 to 1400 ms (*F*s > 5.22), and type on MI trials from 900 to 1099 ms (*F* = 4.68), *p*s < 0.05.

A correlation analysis across subjects explored the relationship between RTs and impoverishment effects at pSW parietal pair 51–52 from 900 1400 ms. Results showed that RT and ERP impoverishment effects from 900 to 1099 ms for real objects correlated significantly for the pSW effect at both 51 and 52 (*r*s > 0.43, *p*s < 0.035). From 1100 to 1400 ms, RT and ERP impoverishment effects for pseudo objects correlated at site 52 (*r* = 0.473, *p* = 0.02). As the pSW became more positive, RTs became slower (Figure 9C).

sLORETA on this data revealed brain sources from 900 ms onwards (Figure 9D) in supplementary motor area (SMA), which was activated in fMRI (Ganis et al., 2007).

### Fragmentation level ERPs

**Figure 10:**
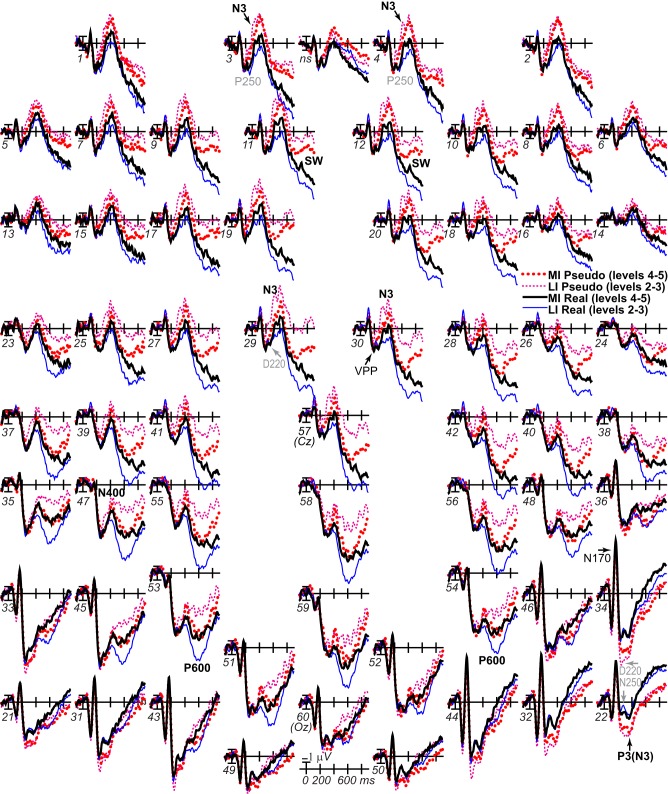
**ERP effects of impoverishment (by fragmentation level) and object type**. Same as Figure 3, except impoverishment is defined using picture fragmentation level: LI is levels 2–3; MI is levels 4–5. Results from this analysis replicate those in Figure 3 which used the median split of RTs to define impoverishment.

The results so far used the median split of RTs to define MI and LI conditions. In a separate analysis of ERPs until 900 ms, fragmentation levels 4–5 defined the MI condition and levels 2–3 defined the LI condition (Figure 10). Results of the fragmentation level analyses replicated all results from the RT split analyses. It may be noted that impoverishment effects for real objects were slightly smaller with fragmentation level defining impoverishment, but this would be expected. After all, the most and least fragmented images were excluded from this fragmentation based analysis but included in the RT based analysis and so stimulus differences were smaller with fragmentation instead of RT defining impoverishment. Further, as RT must completely capture all stimulus impoverishment that affects RTs, impoverishment effects should be larger for results based on RTs than any single factor such as fragmentation.

From 200 to 400 ms, the critical impoverishment by type interaction was found (200–300 ms: x lateral electrode, *F* = 4.5, *p* = 0.002; 300–400 ms, *F* = 16.1, *p* = 0.001; x lateral electrode, *F* = 7.98, *p* <.001; 300–400 ms: midline, *F* = 7.15, *p* = 0.015; x electrode, *F*s > 8.54, *p*s < 0.009), as well as Type and Impoverishment main effects and/or their interactions with scalp site (200–300 ms: *F*s > 3.04, *p*s < 0.02; midline, *F*s > 4.95, *p*s < 0.04; 300–400 ms: *F*s > 2.13, *p*s < 0.04; midline, *F*s > 8.41, *p*s < 0.01). Focal results at frontopolar N3 pair 3–4 showed effects of type (*F*s > 35, *p*s < 0.001) and impoverishment by type (*F*s > 5.07, *p*s < 0.04), and simple effects tests showed impoverishment for real objects from 300 to 400 ms (*F*s > 8.59, *p*s < 0.009), impoverishment for pseudo objects from 200 to 400 ms (*F*s > 4.98, *p*s < 0.04), and type on LI and MI trials from 200 to 400 ms (*F*s > 6.15, *p*s < 0.03). Occipitotemporal pair 21–22 showed type effects from 200 to 400 ms (*F*s > 5.93, *p*s < 0.03), and, from 200 to 300 ms, impoverishment by type (*F* = 5.84, *p* = 0.026). From 300 to 400 ms, the frontocentral N3 (pair 29–30) showed effects of type (*F*s > 6.85, *p*s < 0.02), and impoverishment by type (*F*s > 17.97, *p*s < 0.001), and simple effects tests showed effects of impoverishment for both objects (*F*s > 6.85, *p*s < 0.02), and type on LI trials (*F*s > 25.3, *p*s < 0.001).

From 400 to 700 ms, the critical impoverishment by type interaction was found on the P600 (*F*s > 16.61, *p*s < 0.001; x lateral electrode, *F*s > 2.82, *p*s < 0.02; midline, *F*s > 6.86, *p*s < 0.02; x electrode, *F*s > 5.17, *p*s < 0.03), as well as Type and Impoverishment main effects and their interactions with electrode (*F*s > 4.81, *p*s < 0.05; midline, *F*s > 7.84, *p*s < 0.02). Focal results from 400 to 500 ms showed type and impoverishment main effects at frontopolar pair 3–4 (*F*s > 25, *p*s < 0.01), and a marginal impoverishment by type interaction (*F* = 3.82, *p* = 0.066), and occipitotemporal pair 21–22 showed a type effect (*F* = 15.71, *p* < 0.001). The frontocentral N3 (pair 29–30) showed effects of type (*F*s > 12, *p*s < 0.003), and impoverishment by type (*F*s > 10.79, *p*s < 0.005), and simple effects tests showed impoverishment for real objects, and type for LI trials (*F*s > 4.56, *p*s < 0.048). Focal results from 500 to 700 ms at P600 (pair 55–56) showed effects of type (*F*s > 56.95, *p*s < 0.001), impoverishment by type (*F*s > 25.58, *p*s < 0.001), and type by hemisphere (*F* = 6.97, *p* = 0.017), and simple effects tests showed impoverishment effects for both types, and type on only LI trials (*F*s > 13.22, *p*s < 0.002).

From 700 to 900 ms, the critical impoverishment by type interaction was also found (x lateral electrode, *F*s = 2.62, *p* = 0.044; midline, *F*s = 7.89, *p* = 0.012; x lobe, *F* = 9.9, *p* = 0.006), as well as Type and/or Impoverishment main effects and their interactions with electrode (*F*s > 2.16, *p*s < 0.03; midline, *F*s > 4.88, *p*s < 0.04). Focal results at frontocentral pair 11–12 showed effects of type (*F* = 137.35), *p* < 0.001), and impoverishment by type (*F* = 5.33, *p* = 0.04).

## Discussion

Altogether, a hybrid account that combines the MUSI framework, parietal-prefrontal PHT theories of vision, and decision theories best explains the findings (Table 1 Results). Overall, the ERP time course indicates that knowledge and impoverishment modulate ERPs from 200 to 900 ms, all of which show the impoverished-real-object effect: the N3, centroparietal N400, parietal P600, and a late SW; note, as effects on the LPC were not distinguishable from the P600 and SW, henceforth we do not discuss the LPC. Earlier ERPs and later ERPs from 900 to 1400 ms provide no evidence of this effect, and later ERPs correlate with RTs and reflect supplementary motor cortex activity.

### Early ERPs before 200 ms

Early ERPs are most consistent with the MUSI framework. Early ERPs before 200 ms show no impoverishment nor impoverished-real-object effects. Before 200 ms, there was no evidence that impoverishment affects activation of object knowledge, and the VPP/N170 showed only a small type effect (Table 1). However, a type effect would likely reflect low-level feature differences due to using a subset of pseudo versions of the real objects in order to keep decision rates around 50%; in contrast, prior work compared the full set of matched real and pseudo objects across two experiments and three tasks, finding no ERP differences until after 175 ms and none on the VPP/N170 (Schendan et al., 1998). More important, the VPP/N170 showed no impoverishment effect and no impoverished-real-object effect; note, sensory differences between MI and LI stimuli may have been too small and variable to be detected here. Thus, we found no evidence for early impoverishment effects, and only a small type effect likely reflecting spurious low-level sensory differences due to not using the full set of matched stimuli here. Other studies have also not found early impoverishment effects with these fragmented line drawings, even when level is held constant (e.g., Doniger et al., 2000; Schendan and Kutas, 2002; Schendan and Maher, 2009). With overall no evidence for early impoverishment and type effects independently, it is thus not surprising to find no evidence for an early impoverished-real-object effect. We are thus confident that early effects of impoverishment, type, and their interaction are minimal to none, in general.

Note, early top-down models that involve biasing attention (Figure 1F) assume a cue, context, or target determines task-relevant information, whereas the present task provided no such biasing signal, minimizing such top-down influences early on and consistent with no such evidence here. In real life, context may provide cues about object identity, but, when objects are categorized in scene contexts, similar to real life situations, the N3 complex shows the earliest context effect, not earlier ERPs (Ganis and Kutas, 2003). Possibly only strong, effortful, strategic biased attention would affect early visual processing, as when people visualize features mentally and effortfully before the picture appears, early VPP/N170 modulation is observed (Ganis and Schendan, 2008) and could be expected to be enhanced by impoverishment.

### Late ERPs after 200 ms

Together, both early and later ERPs indicate that object cognition starts after initial bottom-up activation of the ventral stream. Only the MUSI framework, not other vision or decision theories, can explain this pattern. The rest of the discussion thus focuses on later effects and interpretation based on the full ERP time course. While early ERPs best fit the predictions of the MUSI framework, later ERPs best fit the predictions of parietal-prefrontal PHT theories, though MUSI, decision, and prefrontal theories can accommodate the results (Table 1). Thus, a hybrid MUSI account that combines MUSI with parietal-prefrontal PHT and decision theories best explains the findings.

#### Knowledge

Real objects activate knowledge more than pseudo objects so type effects reveal the time course of knowledge activation. The frontal N3, N400, P600, and SW are more negative, and occipitotemporal counterparts of the N3 are more positive for real than pseudo objects, and these effects localize to occipitotemporal cortex. All these ERPs show type effects for LI stimuli, and N3 type effects start at 230 ms for LI stimuli. Altogether, these results replicate evidence for knowledge effects with fully intact (i.e., LI) pictures of real and pseudo objects on these ERPs, starting from 175 to 218 ms during the N3 and continuing onwards (Holcomb and McPherson, 1994; Schendan et al., 1998; McPherson and Holcomb, 1999; Gruber and Müller, 2005, 2006).

While the MUSI account might seem counter to ultra-rapid categorization and other early categorization findings before 150 ms, they are actually compatible. Consider the following. First, for example, eye movement findings (Kirchner and Thorpe, 2006) during ultra rapid categorization suggest an onset at the earliest possible time of around 124 ms when there are more correct than wrong responses. This time matches the 120 ms onset of categorical perception of objects and early object perception processes on the VPP/N170 during State 1 (Schendan et al., 1998; Schendan and Lucia, 2010). When behavior (e.g., a saccade) can be performed based on information from the initial bottom-up pass, then it will be carried out. However, this is a rare occurrence. The same eye movement findings during ultra rapid categorization suggest a mean minimum saccade RT of around 150 ms, and median saccade RT of around 228 ms, varying widely between people, from 159 to 301 ms. Thus while it is tempting to focus on the onset, it is more informative for most visual cognitive phenomena to realize that the fastest times represent a special case of the minimum speed of initial (low-level) visual feed forward processing that is sufficient to enable a decision and motor response (Kirchner and Thorpe, 2006). Most visual input and task situations require more time. Indeed, even ultra rapid categorization tasks pinpoint a typical onset of categorization of 150–228 ms or longer. In addition, longer time (e.g., 150–230 ms or longer) is associated with greater accuracy, even for eye movements, and this additional time is thought to reflect iterative (i.e., interactive, resonant) decision and motor processes (Kirchner and Thorpe, 2006).

Second, as reviewed by Fabre-Thorpe (2011), rapid visual categorization is associated with N3 effects by 150 ms and minimal reaction time (MinRT) of 250 to 300 ms for superordinate categorizations in go-no go and two-category decisions. The shortest time of 220 ms can only be achieved with extensive training, that is, on animal/non-animal decisions with a single overlearned animal scene among novel scenes. No set of easy, trivial, or optimal stimuli can explain this short RT, and MinRT does not shorten even for the simplest geometric images of square vs. circle. However, slower RTs are associated with difficult images, and experience can reduce these RTs (Fabre-Thorpe et al., 2001), consistent with greater repetition priming effects behaviorally and on the N3 and P600/LPC for more than less impoverished categorized objects (Schendan and Kutas, 2003). Altogether, the evidence has led to the conclusion that the role of rapid visual categorization on behavior is limited because it is based on “coarse and unconscious (achromatic) visual representations automatically activated by the first available magnocellular information” that is processed along the ventral visual pathway (Fabre-Thorpe, 2011). Notably, basic level categorization (e.g., dog) yields slightly higher accuracy (4%) than superordinate categorization (e.g., animal), and MinRT is about 50 ms slower (Fabre-Thorpe, 2011). This suggests that, even at the fastest possible time, categorization at the basic relative to superordinate level requires additional processing time, which also achieves a higher decision accuracy. This is consistent with the finding that entry level categorization of new objects is typically associated with an N3 onset time around 200 ms, and repetition priming can reduce this by about 50 ms down to around 150 ms with canonical views, which are not impoverished (Schendan and Kutas, 2003; Schendan and Maher, 2009).

Third, it remains open whether low level feature search can explain the fastest times achieved. Ultra rapid categorization involves giving the subject the category to search for beforehand, making it essentially a visual search task (Treisman, 2006). Hence, before the trial, the visual system has been placed in a “top down presetting” state through feedback processes that prepares it to detect the task relevant features of the category (Enns, 2004; Fabre-Thorpe, 2011). Thus, if a feature of the input matches the top-down search target by 120–150 ms of visual processing, then this can be used to execute a motor behavior (i.e., a saccade), but this does not mean that entry level categorization, meaning, phenomenological awareness, or object cognition has yet occurred. All we know is that a sensori-motor program has been executed within 120 to 150 ms. The MUSI argument is that state 1 may be sufficient for a simple sensori-motor program to be executed based on categorical perception or feature detection (as in visual search), but actual entry level categorization, decision, cognition, and phenomenological awareness do not happen until State 2. What is driving the fastest times in ultra rapid categorization tasks is categorical perception, not actual cognitive categorization. Indeed, it is thought that the 120 ms minimum time for the saccade behavior during ultra rapid categorization tasks may be due to low level visual area V4, bypassing higher order visual areas, such as inferotemporal cortex, sending input directly to lateral inferior parietal cortex and then to frontal eye fields. The earliest 120 to 150 ms times essentially reflect a low level sensorimotor decision that bypasses semantics and even categorical perception, and “is just the start of a series of complex events involving feedback loops…to (generate) conscious perception” (Kirchner and Thorpe, 2006): This is essentially the interactive resonant activity posited in State 2 of the MUSI account.

Fourth, the VPP/N170 and earlier P1 and C1 are thought to reflect predominantly the initial fast feedforward pass through the visual system (e.g., Figure 1A), as well as reflexive feedback (Figures 1B,C), whereas later ERPs are dominated by feedback inputs (David et al., 2005, 2006). Thus feedback has the greatest role in cognition after the initial bottom-up pass.

Fifth, the ERP that shows the earliest effect in ultra-rapid categorization studies is the N3 complex, not the VPP/N170, and across a variety of ultra-rapid categorization studies the N3 is modulated between about 150 and 500 ms (Johnson and Olshausen, 2003, 2005). Interestingly, the onset of the original effect was between 152 and 171 ms (Thorpe et al., 1996). This onset is consistent with ERP findings estimating when entry level categorization starts, that is, between about 150 and 250 ms, modulating the N3. For example, canonical and unusual (impoverished) views of objects differ between 140 and 250 ms (Schendan and Kutas, 2003), repetition effects with canonical (best) views of real objects start to modulate the N3 between 148 and 172 ms (Schendan and Kutas, 2003), and repetition effects for fragmented drawings of real objects that are named correctly start by 192 ms (Schendan and Maher, 2009) or 248 ms (Schendan and Kutas, 2007a). Consistent with the MUSI account, the early part of the N3 effect from 190 to 215 ms on ultra rapid categorization tasks localizes to occipitotemporal cortex (Delorme et al., 2004; Fize et al., 2005), and intracranial ERPs localize ERPs between 200 and 400 ms to VLPFC and occipitotemporal cortex (Allison et al., 1999; Puce et al., 1999).

**Figure 11:**
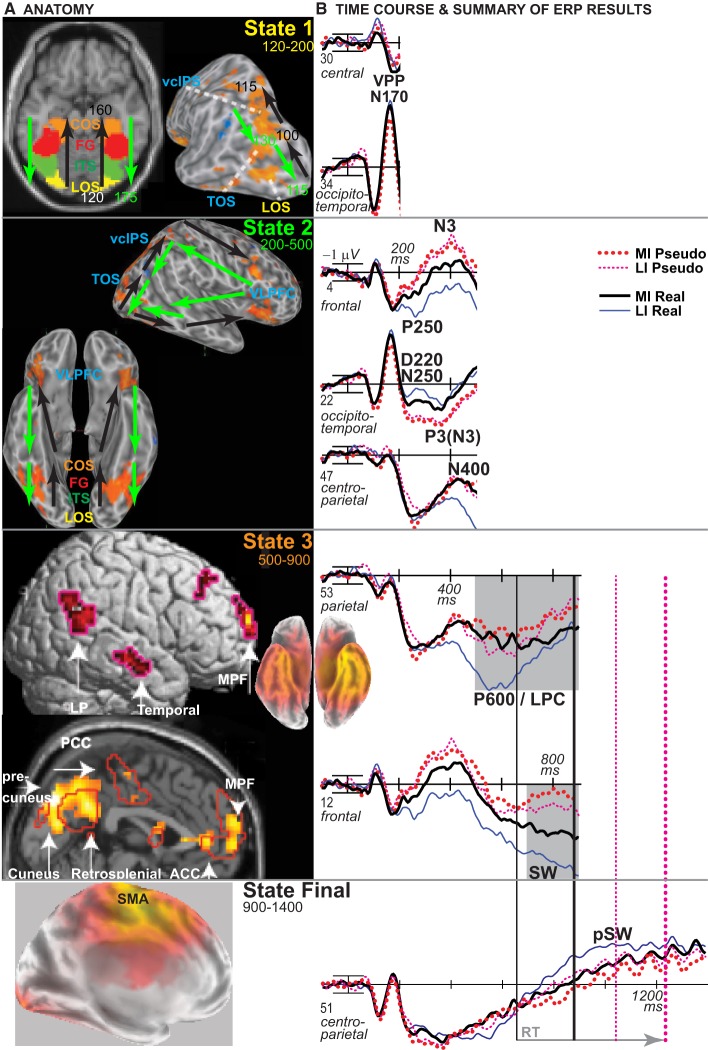
**Multiple-state interactive (MUSI) theory and summary of results**. Numerals indicate time in milliseconds (ms). **(A)** Brain regions contributing to each state of cortical dynamics. Brain images in black box taken from impoverishment effects in fMRI studies of Ganis et al. (2007) for state 2 and dorsal stream of state 1 and Schendan and Stern (2008) for all others. Note: Green arrow is feedback. Black arrow is bottom-up. State 1 axial slice shows object-sensitive areas. VLPFC, ventral lateral prefrontal cortex; vcIPS, ventrocaudal intraparietal sulcus; TOS, transverse occipital sulcus; LOS, lateral occipital sulcus; ITS, inferotemporal sulcus; FG, fusiform gyrus; COS, collateral sulcus; LP, lateral inferior parietal cortex; MPF, medial prefrontal cortex; PCC, posterior cingulate cortex; ACC, anterior cingulate cortex; SMA, supplementary motor area. **(B)** Timing of cortical dynamics and summary of ERP and RT results. In state 1, the VPP/N170 in occipitotemporal cortex (see **A**) shows no impoverished-real-object effect. In state 2, the N3 complex (including N300, P3, P250, D220, N250 components), indexing an interactive network of occipitotemporal, occipitoparietal, and VLPFC regions (see **A**), shows the earliest impoverished-real-object effect. The later N400 also shows such an effect. In state 3, the P600/LPC in temporal lobe parts of a default mode network (see **A**) shows a later impoverished-real-object effect; note, the sLORETA brain source results from Figure 8E are copied here to show the location of P600/LPC effects. The latest such effect modulates an anterior slow wave (SW) in the PCC (see **A**). Gray shading indicates time course of the brain source of the P600 and SW impoverishment effects. A final posterior SW (pSW; state final) correlates with RTs and reflects SMA. Gray arrow points to mean RTs along ERP time course (same legend as for ERPs).

Sixth, the N3 is the first ERP that modulates with categorization success, not the VPP/N170 (Schendan and Kutas, 2002, 2003; Schendan and Maher, 2009). This indicates that object cognition, entry level categorization, and phenomenological awareness of the meaning of the object do not start until feedback interactions dominate processing, especially from anterior temporal or prefrontal cortex down to occipitotemporal cortex, as indexed by the N3 (Lamme, 2003; Schendan and Kutas, 2007a; Folstein and van Petten, 2008; Schendan and Maher, 2009; Clarke et al., 2011). The N3 is more negative with less successful category decisions, greater top-down processes of mental imagery (Schendan and Lucia, 2009; Schendan and Ganis, 2012), greater image atypicality and impoverishment (Doniger et al., 2000; Schendan and Kutas, 2002, 2003; Johnson and Olshausen, 2003), and for new relative to repeated meaningful objects (i.e., repetition priming) (Henson et al., 2004; Schendan and Maher, 2009; Voss et al., 2010). The N3 typically inverts polarity somewhat over occipitotemporal sites, where effects are most prominent with a common average reference (known as N250, Ncl, or L1) and associated with category learning and implicit memory (Gruber and Müller, 2006; Scott et al., 2006; Sehatpour et al., 2006; Soldan et al., 2006). Critically, ERPs and corresponding single-trial EEG and fMRI show that category decision processes that distinguish between faces and objects happen during the N3 complex in state 2 but not on the earlier VPP/N170 in state 1 (Philiastides and Sajda, 2006, 2007; Philiastides et al., 2006; Ratcliff et al., 2009; Rousselet et al., 2011). On functional and spatiotemporal grounds, such work suggests a D220 component of the N3 from 220 to 300 ms (Figures 3, 5A, 9, 11B) varies with impoverishment and task difficulty and reflects anterior cingulate, eye field, insula, and dorsolateral prefrontal activity, and a so-called “late component” of the N3 from 300 to 450 ms reflects decision processes in which VLPFC accumulates evidence from lateral occipital cortex (Philiastides and Sajda, 2006, 2007). For example, the N3 complex and both decision components have similar scalp distribution patterns: Both invert polarity between similar frontal and posterior locations. The role of prediction in visual search (Enns and Lleras, 2008) is consistent with the present finding that interactions or resonance between bottom-up and feedback processes contributes to object constancy and the incorporation of parietal-prefrontal PHT ideas into the MUSI account at state 2.

#### Impoverishment and knowledge

Knowledge activates around 230 ms, and impoverishment and impoverished-real-object effects start around the same time (~250 ms). These onsets are consistent with parietal-prefrontal PHT theory ideas that, when initial bottom-up activation (by ~175–230 ms) cannot categorize the object well enough to make a decision about MI real objects (Serre et al., 2007a), additional processes start to be recruited (~250 ms) that use knowledge in posterior areas to achieve the visual constancy of the category decision. Critically, impoverishment affects real objects the most; note, the flip side of the interaction is that LI stimuli activate knowledge the most effectively. This timing is consistent with the finding from category decision studies of a ~50 ms onset range of single trial EEG discrimination between faces and cars when their phase coherence varies between 30 and 45% (Philiastides and Sajda, 2006). The fMRI and these ERP results are compatible with both (a) top-down processes in the parietal-prefrontal PHT variants (e.g., Ganis et al., 2007) and (b) bottom-up accumulation in decision theories (e.g., Philiastides and Sajda, 2007).

However, only parietal-prefrontal PHT variants predict the interaction (Table 1), and findings from ERP studies of mental imagery indicate top-down processes operate after 200 ms. Mental imagery, which can be mediated only by top-down processes, modulates both the N3 and SW but not the P600 and minimally so the N400 (Schendan and Ganis, 2012). Moreover, ERP mental imagery effects resemble the spatiotemporal characteristics and direction of the impoverishment effects; for example, the N3 and SW are most negative when the need for top-down processes for mental imagery and when impoverishment are greatest. In contrast, adaptation effects, which primarily reflect bottom-up processes, can show ERP effects in the opposite direction to mental imagery and impoverishment effects (Ganis and Schendan, 2008; Schendan and Ganis, 2012). We thus conclude that the N3 impoverished-real-object effect reflects interactive top-down and bottom-up activity that facilitates the category decision because only the N3 reflects visual object knowledge (as argued above) and shows the expected pattern of knowledge, impoverishment, and decision effects across many studies that are predicted by PHT and decision theories.

Accordingly, the N3 impoverishment effects localize to lateral prefrontal cortex (LPFC), and, for real objects only, localize also to the same occipitotemporal region as knowledge activity. This is consistent with the MUSI proposal that the N3 complex reflects interactive activity between VLPFC and occipitotemporal cortex for model selection from object knowledge. After 450 ms, the N400/P600 impoverishment effects for real objects localize to anterior inferior temporal cortex and the mediotemporal lobe, consistent with intracranial studies showing memory effects in anterior mediotemporal lobe that resemble modulations of late posterior positivities on the scalp (Halgren et al., 1995; Guillem et al., 1999; Trautner et al., 2004). After 700 ms, SW impoverishment effects for both object types localize to PCC. As impoverished-real-object effects and their implications change over time, each ERP finding is next discussed in detail separately.

##### N3 complex

The N3 complex shows the earliest impoverishment and impoverished-real-object effects. These findings are consistent with prior evidence of impoverishment or category decision effects on only later ERPs, not at earlier times before ~150–200 ms (Doniger et al., 2000; Schendan and Kutas, 2002, 2003; Johnson and Olshausen, 2003, 2005; Philiastides and Sajda, 2006, 2007; Philiastides et al., 2006; Sehatpour et al., 2006; Ratcliff et al., 2009; Schendan and Maher, 2009; Rousselet et al., 2011).

##### P250/N250 (D220)

The impoverished-real-object effect starts on a frontopolar P250 component of the N3 complex, and this effect inverts polarity occipitotemporally, where it is larger with a common average reference and modulates an N250 over the right hemisphere. At this time, only real objects are more negative frontally and more positive occipitotemporally for MI more than LI stimuli. The P250/N250 indexes processes of model selection from view-specific knowledge acquired based on prior experience categorizing objects at the subordinate level (Schendan and Kutas, 2003, 2007a; Scott et al., 2006). This knowledge also supports entry level categorization (Schendan and Maher, 2009) wherein the decision involves access to semantic memory about meaning (Jolicoeur et al., 1984). The underlying processes have roles in category learning, short-term repetition priming, and working memory, and these ERPs have been found to localize to areas (e.g., lateral occipital cortex) active also during the VPP/N170, consistent with the present source estimation and the MUSI account (Schweinberger et al., 2002; Foxe et al., 2005; Scott et al., 2006; Sehatpour et al., 2006; Ganis and Schendan, 2008; Schendan and Maher, 2009).

The P250/N250 is probably the same as a D220 observed in decision research, as these ERPs have similar time courses and scalp distributions. The D220 modulates with visual impoverishment defined by relative phase coherence of the image and corresponding category decision accuracy, which has been taken as a definition of task difficulty in the diffusion model of decision making (Philiastides et al., 2006). However, the present P250/N250 finding suggests that the D220 also shows the impoverished-real-object effect, arguing against a generic task difficulty interpretation. Instead, the P250/N250 (D220) reflects the interaction between decision processes, visual perception, and memory (i.e., category knowledge). If the D220 was related only to task difficulty, then it should also show an impoverishment effect for pseudo-objects, which it does not. In addition, the D220 is specific for decisions about the object's category as opposed to its color or episodic familiarity (Philiastides et al., 2006; Schendan and Lucia, submitted) so access to category knowledge is an integral part of the underlying neural processes. Relative to category decisions, color decisions were considered easier (Philiastides et al., 2006), but episodic recognition takes longer and so can be considered harder. Nonetheless, the N3 complex, including the D220, shows an impoverishment effect for category more than episodic memory decisions (Schendan and Lucia, submitted). Further, color decisions do not automatically activate category knowledge (Boucart and Humphreys, 1994; Pins et al., 2004). Thus knowledge activation, not task difficulty, explains why the D220 disappears when the task is color decision.

##### N3 significance

Altogether, the findings on the N3 complex indicate that PHT and decision processes start around 250 ms (i.e., the onset of when impoverishment affects knowledge activation) and lasts until around 500 ms post-stimulus onset. Impoverishment affects processing earlier for real than pseudo objects. For real objects, impoverishment makes the frontal N3 complex more negative from 250 to 450 ms, whereas, pseudo objects show no such effect. These N3 findings are consistent with previous work indicating that the frontopolar N3 varies with the success of categorization and degree of mental rotation (Schendan and Lucia, 2009; Schendan and Maher, 2009). They are also consistent with the idea that the underlying process primarily detects the relative match to stored information. Evidence indicates that the N3 complex indexes model selection from object information in occipitotemporal cortex based on the relative similarity of the shapes and parts in a specific view, regardless of the constituent small line segments, and working memory and long-term perceptual priming modulate these processes (Holcomb and McPherson, 1994; McPherson and Holcomb, 1999; Doniger et al., 2000, 2001; Daffner et al., 2000b; Schendan and Kutas, 2002, 2003, 2007a; Henson et al., 2004; Gruber and Müller, 2006; Sehatpour et al., 2006; Soldan et al., 2006; Ganis and Schendan, 2008). The neurophysiological processes underlying the N3, perhaps especially frontopolar components, likely contribute critically to processes of *similarity* evaluation for visual object cognition. Testing processes in PHT theories require evaluating the similarity of the spatial configuration (i.e., location) of features between object representations (e.g., between a predicted model and a perceived object). After all, shape similarity drives neural responses in monkey inferotemporal and human occipitotemporal cortex, and is important for category learning (Li et al., 1993; Rainer and Miller, 2000; Freedman et al., 2001, 2002, 2003; Sigala and Logothetis, 2002; Sigala et al., 2002; Sigala, 2004; Jiang et al., 2007; Kriegeskorte et al., 2008; Op de Beeck et al., 2008). Further, most categorization theories posit a central role for evaluation of similarity, especially perceptual similarity acquired through perceptual learning (Goldstone, 1994; Kruschke, 2008), and perceptual learning depends upon processing to a point at which perceptual constancy is achieved (Garrigan and Kellman, 2008).

##### N3 brain sources

Cortical source findings indicate that LPFC and occipitotemporal cortex activate together during the N3 and the posterior contribution includes knowledge-related processing, consistent with top-down parietal-prefrontal PHT, decision, and MUSI theories. While N3 impoverishment effects localize to the LPFC, regardless of knowledge, they also localize to occipitotemporal cortex only for real objects from 255 to ~450 ms. By a PHT account, impoverishment of real objects recruits LPFC, which can succeed in modulating object knowledge stored in occipitotemporal cortex, resulting in an impoverishment effect there as well. Impoverishment of pseudo objects also recruits LPFC, but this has little or no modulatory influence on occipitotemporal activity because, by design, these unknown images activate knowledge minimally if at all. Intracranial ERPs extracted from LPFC and occipitotemporal sources show that these impoverished-real-object effects start only after the bottom-up pass (after ~200 ms). While source estimates are inherently uncertain due to the inverse problem, our localizations fit the areas showing impoverished-real-object effects in fMRI (Ganis et al., 2007; Schendan and Stern, 2008) and are far distant from each other and so spatially resolvable (Pascual-Marqui, 2002; Wagner et al., 2004).

##### N400, P600, SW

Knowledge modulates the N3 and SW with both LI and MI stimuli but the N400 and P600 only with LI stimuli. Because subjects must activate knowledge in order to make a category decision with both LI and MI stimuli, this finding pinpoints the N3 and SW as candidates for reflecting the critical knowledge activity. However, the anterior SW does not differ between MI unusual and LI canonical views (Schendan and Kutas, 2003) and so is not a general impoverishment marker, and the SW does not show repetition effects with categorized real objects, as it should if it reflects memory (Schendan and Maher, 2009). Thus, the N3 is only viable candidate for a neurophysiological marker of PHT and decision processes that mediate the impoverished-real-object effect.

Only ERPs from 400 to 700 ms show a knowledge (type) effect only for LI stimuli. Thus, during the N400 and P600, underlying semantic memory and decision evaluation processes, respectively, take place for LI but not MI stimuli. In contrast, the earlier N3 and later SW show knowledge effects at both impoverishment levels, though more for LI than MI, dissociating late ERPs from each other. This dissociation between the N3 and N400/P600 supports a dichotomy (Kousta et al., 2011) between experiential (sensorimotor, affect) knowledge, as indexed by the N3 for vision, and linguistic (verbal) knowledge, indexed by the N400, and later strategic evaluation of earlier category decision processes and secondary higher-order semantic memory analysis, indexed by the P600/LPC (Schendan and Kutas, 2002; Sitnikova et al., 2010).

While all later ERPs after 200 ms show the impoverished-real-object effect, the exact pattern of the interaction differs, dissociating the meaning of these effects. The N3 and N400 findings indicate that LI images of real objects activate knowledge, including meaning, more strongly than MI images of them. After all, the N3 and N400 show impoverishment effects for real objects only. In contrast, the P600 and SW show impoverishment effects also for pseudo objects. Indeed, impoverishment affects processing of pseudo objects for the first time only later, after 500 ms on the P600 and SW. As impoverishment effects apply also to pseudo objects, which cannot activate knowledge, this suggests that these latest effects to some extent reflect response related processes after the category decision. Consistent with this, the P600 seems to index evaluating how well or confidently a task goal or memory matching process has succeeded (Ruchkin and Sutton, 1978; Schendan and Maher, 2009). The P600 is larger on LI than MI trials because LI stimuli are more confidently categorized than MI stimuli, enhancing the P600 and related to faster RTs for LI than MI stimuli. Accordingly, source findings indicate that impoverished-real-object effects during the P600 reflect post-categorization processes in anterior inferior and mediotemporal cortex related to evaluating the decision and memory match, and, after 700 ms during the SW, response planning related processes in a PCC region. These regions show impoverished-real-object effects in fMRI, though PCC shows deactivation (i.e., more active for LI than MI) (Ganis et al., 2007; Schendan and Stern, 2008). Altogether, the ERP time course indicates that larger fMRI impoverishment activations for real (than pseudo) objects reflect both earlier processes during the N3 and N400 and later processes during the P600 and SW, whereas the smaller fMRI impoverishment activations for pseudo objects reflect only later processes after 500 ms.

##### N400 linguistic knowledge

From 400 to 500 ms, impoverishment modulates the centroparietal N400, which is smallest for LI real objects relative to all other conditions. The idea that name, semantic (i.e., indexed by N400), and object model (or “structural description,” i.e., indexed by the N3) knowledge interact bidirectionally to achieve visual object categorization and naming is consistent with an interactive activation and competition model of object naming (Humphreys et al., 1999) and the MUSI account. By such accounts, the present finding of an impoverished-real-object effect on the N400 would indicate that interactive computations among knowledge systems, including linguistic semantic memory, also have a role in achieving visual constancy of the cognitive decision. However, source findings suggest only posterior contributions from occipitotemporal and anterotemporal cortex. As no evidence was found for prefrontal-posterior interactions during the N400, word-related semantic memory may not contribute to perceptual hypothesis testing but rather activates after the category decision.

##### P600

By the MUSI account, the P600 in state 3 reflects strategic evaluation. P600 (or LPC) knowledge effects may also in part reflect stimulus categorization (Dien et al., 2004). The ~50% overall categorization rate confirms that the ERP effects do not reflect differences in subjective probability of categorization success associated with P3(00)-like ERPs (Polich and Bondurant, 1997). The P600 shows impoverishment effects for the first time for both real and pseudo objects. The P600 is more positive for LI than MI stimuli for real more than pseudo objects. The P600 effect for real objects replicates the finding that the P600 is larger to LI canonical than MI unusual views on categorization and recognition (Schendan and Kutas, 2003; Schendan and Lucia, submitted).

##### SW

After 700 ms, a broadly distributed SW with a midline central maximum differs among all conditions. The SW impoverished-real-object effect manifests as greater positivity for LI real objects relative to MI ones relative to LI pseudo objects relative to MI ones. The SW seems to index processes related to response execution and monitoring, being less positive when these processes are more challenging (Schendan and Maher, 2009). After 700 ms during the SW, impoverishment effects localize primarily to the PCC region that instead activates more for LI than MI real objects in fMRI (Ganis et al., 2007; Schendan and Stern, 2008). The PCC is part of a default mode network for internal evaluation, exogenous attention, episodic memory retrieval, and semantic memory computations with words that is anticorrelated in fMRI with the active task network that instead includes prefrontal and posterior processing areas that underlie the N3 and N400 (Fox et al., 2005; Buckner et al., 2008; Binder et al., 2009). The present time course would be consistent with the idea that the active task network operates from 200 to 500 ms during the N3 and N400, whereas the P600 and SW reflect activity in the mediotemporal and PCC parts of the default mode network, respectively. Intriguingly, after 700 ms, real objects activate anterior and medial temporal cortex and PCC, whereas pseudo objects activate only the PCC. This suggests that knowledge in temporal cortex contributes to PCC activity as part of default mode interactions with real objects but not pseudo objects, which cannot activate knowledge. Because the anterior and medial temporal cortex activity starts during the P600, the same activity during the later SW likely reflects a continuation of the earlier posterior positivity and may best be considered an LPC contribution to posterior ERPs after 500 ms (P600, SW).

### Alternative explanations

#### Not subjective probability

Subjective probability of categorized vs. uncategorized responses cannot explain the results. Subjects were naïve that some objects were not real (pseudo-objects) and so uncategorizable, and categorized and uncategorized responses split about evenly: From the subjects' perspectives, any object, whether truly real or pseudo, that did not belong to a known category was merely an uncategorized object, and this happened about half the time, making the task essentially a reliable and simple two-choice decision between half categorized and half uncategorized images.

#### Not early motor potentials

N3 effects do not reflect earlier time courses of motor potentials for LI than MI objects. (a) The N3 and RTs dissociate. The N3 complex shows impoverished-real-object effects well before the earliest RT to LI real objects (~650 ms). Still, if the N3 is merely a motor potential, a larger N3 should always be associated with longer RTs. To the contrary, it has been found that, when people categorize fragmented pictures of objects that have been repeated (primed), the N3 is the same between all repetition conditions, whereas RTs and other ERPs, such as the P600, differ between the various repeated conditions (Schendan and Kutas, 2007a,b). Further, the N3 is larger when categorization RTs are faster (instead of slower) for scrambled than intact objects (Schendan and Lucia, 2010). (b) The N3 does not index a motor readiness potential. The readiness potential (RP) is a midline central negativity that is greater for contralateral than ipsilateral responses by ~200 ms post-stimulus due to differential activity in primary motor cortex. The RP could make negativity greater for MI than LI stimuli but cannot explain these N3 effects. First, with a mastoid reference, as herein, the RP is maximal over central midline (C3, C4) and absent at frontal sites (F3, F4) (Kutas and Donchin, 1980) near the frontocentral N3 and far from the frontopolar ERPs. Second, N3 and RP waveforms differ. The N3 impoverishment effect and its LPFC sources return to baseline by 500 ms, which is ~150 ms before the earliest RT. In contrast, the RP rises steadily over ~500 ms preceding the RT (Coles, 1989). Third, no impoverishment effects were found in primary motor cortex in our N3 source estimates and neuroimaging studies of model verification (Kosslyn et al., 1994; Ganis et al., 2007; Schendan and Stern, 2008). (c) N3 impoverishment effects cannot merely be related to motor planning. An impoverishment effect in the supplementary motor area was found in the fMRI version for fragmented pictures (Ganis et al., 2007) but not for unusual vs. canonical views (Kosslyn et al., 1994; Schendan and Stern, 2008). Only ventral premotor cortex activity reflects a general process related to image impoverishment with objects (unusual views, fragmented pictures) (Ganis et al., 2007; Schendan and Stern, 2008) that has been implicated in evidence accumulation for a decision (Heekeren et al., 2008). Finally, note that N3 knowledge effects are unlikely to reflect differences in motor responses between real and pseudo objects because similarly large N3 differences have been found with the full set of these stimuli during passive viewing when both object types were non-targets (Schendan et al., 1998), but P600 (or LPC) knowledge effects may in part reflect stimulus categorization (Dien et al., 2004).

#### Late motor activity (pSW)

The most likely ERPs to include motor potentials are those around the time of the response. Indeed, after 900 ms, a posterior slow wave (pSW) (Figure 9) modulates independently with type and impoverishment but shows no evidence of impoverished-real-object effects. The pSW is more positive for real than pseudo objects and for MI than LI trials, matches and correlates with corresponding RT effects, and localizes to SMA and nearby anterior cingulate regions that show impoverishment effects in fMRI (Ganis et al., 2007). These sources are consistent with late slow intracranial ERPs in premotor and motor regions of epilepsy patients (Halgren et al., 1994). However, the SMA region and pSW findings do not reflect model verification *per se* but rather later processes related to generating a response under MI relative to LI conditions because it was not specific for real objects, and SMA shows no effects of impoverishment by viewpoint (Schendan and Stern, 2008).

#### Nonvisual impoverishment factors

The median split approach captures all possible factors that contribute to the visual constancy of a category decision, but using fragmentation level to define LI and MI conditions yields the same pattern (Figure 10), demonstrating that visuoperceptual factors were among those driving the effects. Further, impoverishment effects here resemble those found when fragmentation or viewpoint impoverishes the images (Doniger et al., 2000; Schendan and Kutas, 2003). Future work will need to tease apart each perceptual and cognitive factor using the times and regions of interest defined here.

## Conclusion

Findings reveal the cortical dynamics to achieve visual constancy of a category decision. The time course of knowledge, impoverishment, and impoverished-real-object findings fit best a hybrid MUSI account that incorporates parietal-prefrontal PHT theories and decision theories to explain the visual constancy of object cognition. By such an account, for MI objects, the initial bottom-up pass may fail to yield a sufficiently accurate decision, thereby recruiting prefrontal cortex to send top-down modulatory inputs to occipitotemporal object processing areas to accumulate more perceptual and knowledge evidence for the decision. Critically, by examining both impoverishment and knowledge factors, the findings demonstrate that impoverishment adversely affects activation of knowledge (conveyed by real objects) more than merely perceptual processing of any object (including pseudo objects) by ~250 ms after seeing an image. Convergent evidence, including from studies of the top-down processes for mental imagery, lead to the conclusion that, during the N3 complex, top-down processes posited in parietal-prefrontal PHT and decision theories recruit LPFC to modulate not only perceptual evidence coming from posterior object processing areas but also the activation of knowledge in those areas. This happens after the initial bottom-up activation of object processing areas.

Altogether these findings suggest the following hybrid MUSI account, which incorporates parietal-prefrontal PHT and decision theories, to explain the cortical dynamics for the visual constancy of object cognition (Figure 11). State 1 during the VPP/N170 between 120 and 200 ms involves initial, bottom-up activation of ventral object processing cortex. Starting ~230 ms (state 2), model selection based on both visual input and memory (e.g., knowledge) for a decision starts during a second state of interactive bottom-up, recurrent, and feedback (reflexive top-down) activity among object processing areas in occipitotemporal cortex and VLPFC, indexed by the N3 complex. When visual input is highly impoverished, top-down processes of PHT in parietal and LPFC areas, especially VLPFC regions, can modulate occipitotemporal activity to facilitate the visual object constancy of a decision, achieving accuracy at a cost of longer response times. Any impoverished image can recruit PHT processes, but these processes modulate knowledge-related computations in occipitotemporal cortex only when the image depicts a real object. Based on convergent evidence, we propose that top-down processes for PHT are recruited based on the shape similarity among perceived object(s) and stored models (i.e., match between percept and knowledge), which decreases as image impoverishment increases. Also in state 2, during a centroparietal N400 from 400 to 500 ms, interactive activation of linguistic (verbal) knowledge (e.g., the name) happens in temporal cortex. Later after ~500 ms (state 3), anterotemporal cortex during the P600/LPC and posterior cingulate activity during a broad slow wave (SW), perhaps in the default mode network for internal evaluation of prior processing and memory activation, and secondary higher-order semantic memory. Finally, after 900 ms (in a final response state), SMA and anterior cingulate activity, indexed by a posterior slow wave correlated with RTs, plans the execution of the motor response.

## Funding

Research supported by Research Executive Agency European Union, Seventh Framework Programme (FP7), Marie Curie Career Integration Grant: PCIG09-GA-2011-294144-COGNITSIMS, the Research Executive Agency European Union FP7 Marie Curie Initial Training Networks (ITN) FP7-PEOPLE-2013-ITN-604764 Innovative Doctoral Programme (IDP): COGNOVO, and Plymouth University Grants to HS from the Social Science Collaboration with the University of Exeter Scheme, the International Research, Networking and Collaboration Grant, and the Faculty Innovation Centre (FInC) Grant.

### Conflict of interest statement

The authors declare that the research was conducted in the absence of any commercial or financial relationships that could be construed as a potential conflict of interest.
